# Structure-functional characterization of *Lactococcus* AbiA phage defense system

**DOI:** 10.1093/nar/gkae230

**Published:** 2024-04-08

**Authors:** Marta Gapińska, Weronika Zajko, Krzysztof Skowronek, Małgorzata Figiel, Paweł S Krawczyk, Artyom A Egorov, Andrzej Dziembowski, Marcus J O Johansson, Marcin Nowotny

**Affiliations:** Laboratory of Protein Structure, International Institute of Molecular and Cell Biology, Warsaw, Poland; Laboratory of Protein Structure, International Institute of Molecular and Cell Biology, Warsaw, Poland; Biophysics Core Facility, International Institute of Molecular and Cell Biology, Warsaw, Poland; Laboratory of Protein Structure, International Institute of Molecular and Cell Biology, Warsaw, Poland; Laboratory of RNA Biology, International Institute of Molecular and Cell Biology, Warsaw, Poland; Department of Experimental Medical Science, Lund University, 221 00 Lund, Sweden; Laboratory of RNA Biology, International Institute of Molecular and Cell Biology, Warsaw, Poland; Department of Experimental Medical Science, Lund University, 221 00 Lund, Sweden; Laboratory of Protein Structure, International Institute of Molecular and Cell Biology, Warsaw, Poland

## Abstract

Bacterial reverse transcriptases (RTs) are a large and diverse enzyme family. AbiA, AbiK and Abi-P2 are abortive infection system (Abi) RTs that mediate defense against bacteriophages. What sets Abi RTs apart from other RT enzymes is their ability to synthesize long DNA products of random sequences in a template- and primer-independent manner. Structures of AbiK and Abi-P2 representatives have recently been determined, but there are no structural data available for AbiA. Here, we report the crystal structure of *Lactococcus* AbiA polymerase in complex with a single-stranded polymerization product. AbiA comprises three domains: an RT-like domain, a helical domain that is typical for Abi polymerases, and a higher eukaryotes and prokaryotes nucleotide-binding (HEPN) domain that is common for many antiviral proteins. AbiA forms a dimer that distinguishes it from AbiK and Abi-P2, which form trimers/hexamers. We show the DNA polymerase activity of AbiA in an *in vitro* assay and demonstrate that it requires the presence of the HEPN domain which is enzymatically inactive. We validate our biochemical and structural results *in vivo* through bacteriophage infection assays. Finally, our *in vivo* results suggest that AbiA-mediated phage defense may not rely on AbiA-mediated cell death.

## Graphical Abstract

**Graphical Abstract:**
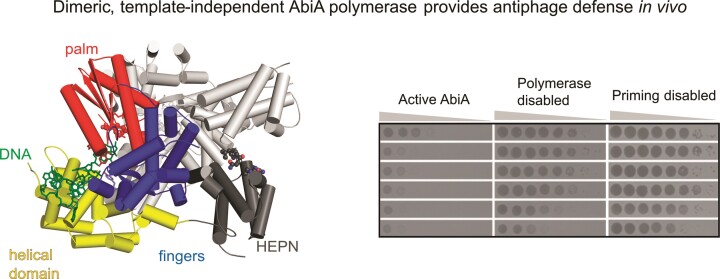

## Introduction

Bacteriophages—or simply phages—are viruses that infect bacteria. Phages and their prey are engaged in a constant arms race. To resist predation by phages, bacteria employ diverse defense mechanisms, which phages counter with ingenious anti-defenses (1). Bacteria tend to encode phage defense systems on so-called defense islands of the chromosome (2,3). Furthermore, defense systems are also often encoded by bacterial Mobile Genetic Elements (MGEs). Lysogenic phages—prophages—encode multiple antiphage systems with a purpose of rendering the bacterial host immune to superinfection with other phages and, therefore, giving the prophage a competitive advantage (4). Similarly, plasmids also often encode antiphage defenses that contribute to increased evolutionary success of plasmid-carrying strains (5).

Defense systems act in concert, collectively forming bacterial immune system (6). The first line of defense aims to inhibit or limit phage adsorption, thus precluding the viral infection altogether. This kind of systems are exemplified by twitching inhibitory protein (Tip) (7). Tip binds and inhibits the type IV pilus assembly protein PilB, which in turn compromises the formation of pili, common phage receptors. The next line of defense is exemplified by systems that recognize and destroy phage DNA once it was injected in the cell; notable examples are CRISPR-Cas (8) and restriction–modification (R–M) systems (9). Finally, if all these systems are breached, bacteria employ the last-ditch defense tactics: abortive infection systems. Upon detecting the phage infection, these systems cause bacterial cell death or dormancy, thus preventing the phage from completing its replication cycle and spreading in the bacterial population (10,11). Even if the individual infected bacterium dies, this defense strategy prevents the rest of the colony from being exposed to the infection (11). Abortive defense systems can be triggered by pathogen-associated molecular patterns (PAMPs), such as structural proteins (12,13). Alternatively, they can be turned on by sensing the inhibition of bacterial CRISPR and R–M systems by phage-encoded factors, as exemplified by PARIS (14) and PrrC (15).

Prokaryotic reverse transcriptases (RTs) are a diverse group of enzymes, with several subfamilies being involved in antiphage defense: Abortive infection (Abi) RTs, CRISPR-Cas-associated RTs, unknown RTs (UG) (16), and defense-associated RTs (DRTs) (17). The three well-studied Abi RT representatives are AbiK, AbiA and Abi-P2. AbiK was initially discovered as a plasmid-encoded defense system that protects *Lactococcus lactis* strain W1 from diverse *Siphovirus* morphotype phages: P335, 936 and c2 (18). AbiK proteins are widespread among *Lactobacillales*, *Bacillales* and *Eubacteriales* (19). Similarly to AbiK, AbiA was also initially discovered as a plasmid-encoded antiphage defense system in *L. lactis* (20). AbiA provides *L. lactis* resistance against phages c2, *Skunavirus* p2, sk1 and φ31 (21). The *abiA* gene is constitutively transcribed, and an increase in the copy number translates into increased efficiency of the antiviral activity (21). Lactococcal AbiA expressed in *S. thermophilus* grants resistance against phages Q1, Q8, Q9, 1FN and 2FN (22). Finally, Abi-P2, first discovered in an *E. coli* P2-like prophage, was shown to provide resistance to phage T5 which belongs to the *Demerecviridae* family (23).

While based on sequence homology AbiA, AbiK and Abi-P2 enzymes are classified as RTs, their enzymatic activity resembles that of terminal transferases (24). AbiK has been shown to synthesize long DNA strands of seemingly random sequence, without a primer or template (24). Priming occurs by attachment of the first nucleotide to a hydroxyl group of one of the tyrosine residues in the protein (25). Structures of AbiK and Abi-P2 proteins were determined recently, revealing their unusual trimeric/hexameric configuration (25). However, no structural or mechanistic information is available for AbiA. The exact mechanism of defense mediated by Abi RTs is currently unclear. Intriguingly, the results presented in a recent PhD thesis by A. Du suggested that *Lactococcus* AbiK might defend against the phage attack without necessarily causing the death of the infected cell (26), thus suggesting that Abi systems might not all necessarily be abortive infection systems *sensu stricto*.

Here, we present a crystal structure of AbiA. The protein contains three domains: an RT-like polymerase domain, a helical domain observed so far only for Abi RTs and predicted for UG RTs (17,27) and a C-terminal higher eukaryotes and prokaryotes nucleotide-binding domain (HEPN) domain, which is widespread among antiviral defense systems, such as toxin–antitoxin (28) and CRISPR-Cas (29). Unlike AbiK and Abi-P2 polymerases, AbiA forms dimers. We confirm the template-independent polymerase activity in AbiA and show that the HEPN domain, which mediates AbiA dimerization, is important in this process. Finally, we demonstrate that *Lactococcus* AbiA and AbiK are active in phage defense in an *E. coli* surrogate host. Through mutational analysis we support the biochemical results in experimental infection assays. By performing infection assays with increasing Multiplicity Of Infection (MOI) we provide *in vivo* evidence suggesting that AbiA-mediated phage defense does not rely on AbiA-mediated cell death.

## Materials and methods

### Reagents and biological resources

*Reagents*. For crystallization, we used the PEGRx screen (Hampton Research). For the biochemical experiments, we used α-^33^P-dATP 3000 Ci/mmol, 10 mCi/ml, 250 uCi, Hartman Analytics.

*Biological resources*. The present study used the following *E. coli* strains: BL21 Star (DE3) (F^−^*ompT hsdSB* [r_B_^−^ m_B_^−^] *gal dcm*^+^*rne131* [DE3]), BL21 Gold (DE3) (B F^−^*ompT hsdS* [r_B_^−^ m_B_^−^] *dcm*^+^ Tet^r^*gal* alt hsdS*endA* Hte), Top10 (F^−^*mcrA* crA*mrr-hsdRMS-mcrBC]* rr-*lacZ*Δa15 Δ*lacX74 recA1 araD139* ac*ara-leu*]*7697 galU galK rpsL* [Str^r^] *endA1 nupG*) and BW25113 K-12 strain (F^−^, *Δ(araD-araB)567, ΔlacZ4787(::rrnB-3), λ*^−^, *rph-1, Δ(rhaD-rhaB)568, hsdR514)*.

### Plasmid construction

For expression in *E. coli*, a synthetic gene that encodes the full-length AbiA protein from *L. lactis*, subcloned into a pET28a–small ubiquitin-like modifier (SUMO) expression vector, was obtained from BioBasic. The vector contained an N-terminal His_6_-SUMO-tag, removable by SUMO protease. A variant of this expression construct that contained only an N-terminal His_6_-tag was prepared by sequence-ligation independent (SLIC) cloning method and was used to introduce point amino acid substitutions using a site-directed mutagenesis approach. The construct for expression of His_6_-SUMO-tagged AbiA without the HEPN domain (ΔHEPN, residues 1–478) was prepared by introducing a stop codon.

For phage immunity assays, the *abiA* and *abiK* genes were cloned under the P_tet_ promoter in plasmid pJD1423 (VHp1423), which a pBR322 derivative where the *tetA* open reading frame (ORF) has been replaced by the *rrnB* terminator (30). The pJD1423-*abiA* (VHp1642) and pJD1423-*abiK* (VHp1639) plasmids were constructed by Gibson assembly (31). In these plasmids, the *abiA* and *abiK* ORFs are preceded by the sequence for a strong Shine- Dalgarno element (5′-AGGAGGAATTAA-3′). Mutant variants of AbiA were constructed by site-directed mutagenesis of VHp1642, generating pJD1423-*abiA^D240N^* (VHp1661), pJD1423-*abiA^R581A^* (VHp1662), pJD1423-*abiA^H588A^* (VHp1663), pJD1423-*abiA^R581A H588A^* (VHp1664), pJD1423-*abiA^Y298F^* (VHp1665), pJD1423-*abiA^Y303F^* (VHp1666), and pJD1423-*abiA^Y298F Y303F^* (VHp1667). All constructs were verified by DNA sequencing.

### Expression and purification of AbiA

The recombinant protein was produced in *E. coli* BL21 STAR cells. The bacterial pellet was suspended in a buffer that consisted of 50 mM Tris (pH 7.0), 150 mM NaCl, 20 mM imidazole, 5% glycerol and 5 mM 2-mercaptoethanol. It was incubated on ice with a mixture of protease inhibitors, lysozyme, and viscolase (A&A Biotechnology). After sonication, the cleared lysate was loaded onto a HisTrap HP column (GE Healthcare) that was equilibrated with a buffer that contained 20 mM imidazole, 25 mM Tris (pH 7.0), 0.5 M NaCl, 5% glycerol, and 5 mM 2-mercaptoethanol. After a wash with 150 mM imidazole, the protein was eluted with 400 mM imidazole. SUMO protease was added to the protein fraction to remove the tag, and then the sample was dialyzed overnight against a buffer that contained 20 mM imidazole. The protein sample after dialysis was analyzed on a sodium dodecyl sulfate-polyacrylamide (SDS-PAGE) gel, but a band shift indicative of a lower protein molecular weight that resulted from SUMO removal was not visible. After reapplying this fraction to the HisTrap column, the protein was again eluted in fractions with high imidazole concentrations, confirming that the SUMO tag was not cleaved off by the protease. The fraction that contained SUMO-AbiA was concentrated and applied to a Superdex 200 column. The protein was eluted as a single symmetrical peak, with a volume that corresponded to a molecular weight of ∼150 kDa. A variant of AbiA that contained only an N-terminal His_6_-tag was purified according to the same protocol, but without SUMO protease addition step.

### Protein crystallization

A purified AbiA protein without SUMO tag was initially used for crystallization. It did not, however, produce any crystals. On the contrary, AbiA with an uncleaved SUMO tag crystallized in a wide range of concentrations and conditions. Because of this, we continued the crystallization experiments with this protein variant. Protein crystals were grown in the PEGRx screen (Hampton Research) and further optimized manually. The following crystallization robots were used: Phoenix (Art Robbins Instruments, Sunnyvale, CA, USA) and Oryx (Douglas Instruments, Hungerford, UK). Crystals that diffracted X-rays to the highest resolution were obtained at a protein concentration of 5 mg/ml under the following conditions: 0.2 M sodium potassium tartrate tetrahydrate, 0.1 M Bis–Tris (pH 6.0), and 9% PEG 10 000. For crystallization, both the hanging-drop and sitting-drop vapor diffusion methods were used at 18°C. For data collection, the crystals were flash frozen in liquid nitrogen.

### Data collection and structure determination

The X-ray diffraction data for SUMO-AbiA were collected at the 14.1 beamline at the BESSY synchrotron (Berlin, Germany) (32). The best dataset extended to approximately 2.8 Å resolution and was collected at a wavelength of 0.9184 Å using the Pilatus detector. The crystals of SUMO-AbiA belonged to the *P*1 space group, and the diffraction data were processed using XDS software (33). The structure was solved by molecular replacement with Phenix Phaser-MR software using the model that was generated by AlphaFold 2 (34). Automatic model refinement was performed using phenix.refine (35), and manual amino acid chain building was performed using Coot (36). The asymmetric unit of the crystal contained four AbiA molecules that formed two dimers. Of the 628 amino acid residues of each protomer, all were visible except loops that comprised amino acids 290–310, 450–456 and 587–597. The SUMO tag was not visible in the structure. In the structure, we observed additional electron densities, corresponding to 5–9 nucleotide (nt) fragments of single-stranded DNA (ssDNA). Template-free DNA synthesis, conducted by Abi polymerases, is known to result in random sequences (24). Therefore, in the AbiA structure, we built DNA chains with arbitrarily chosen sequences.

### Activity assays

Assays of AbiA template-independent DNA polymerization were initially conducted using Texas Red-dCTP. However, this fluorescent nucleotide yielded products of two well-defined sizes rather than a ladder pattern. We concluded that these were not natural products of the DNA polymerization and in the next round of experiments we used α-^33^P-dATP. In the assays we used AbiA proteins with N-terminal His-tag (wild type and point substitution variants). The reaction buffer contained 50 mM Tris (pH 7.0), 250 mM NaCl, 10 mM MgCl_2_, and 5 mM dithiothreitol (DTT). To radiolabel primers copurified with the protein, AbiA (0.5 μM) was incubated with 5 μCi (0.16 μM) of α-^33^P-dATP (Hartmann Analytic) at 37°C for 1 minute in the presence of the reaction buffer. The labeling mix was diluted fourfold in the reaction buffer containing equimolar mix of 150 μM of all four deoxynucleotide triphosphates. Aliquots of primer extension reactions were collected at 1.5, 3 and 4.5 min and added to a stop mix containing EDTA and proteinase K. To test the deoxynucleotide preference of AbiA polymerase activity, the labeling mix was diluted twofold in the reaction buffer containing 200 μM of either dGTP, dATP, dTTP or dCTP. Primer extension reactions were stopped after 4.5 min. Reaction products were analyzed on 15% TBE-urea polyacrylamide gels and visualized by autoradiography. Molecular size markers (20, 50, 65 and 97 nt radiolabeled at 5′ end with polynucleotide kinase) were used to assess the size of the polymerization products. All reactions were performed in duplicate. Densitometry analysis was performed on the gels. Bands that corresponded to DNA products were quantified with ImageQuant software (Cytiva).

For external primer extension assay, 0.5 μM Cy5-5′-labeled primer:

5′-AATAAACACCACGTGTGA-3′ was incubated at 37°C for 15 min with 0.1 μM AbiA Y298F/Y303F variant. Then dNTP mix was added at a concentration of 250 μM, the reaction was further incubated for 1, 10 and 15 min. Reaction was then stopped by the addition of 40 mM EDTA and Proteinase K. Reaction was also performed in the presence of a duplex which contained the same labeled DNA primer with DNA (GTCAGTGTGTTAATCTTACAACCAGAACTCAATTACCCCCTGCATACACTAATTCTTTCACACGTGGTGTTTATT) or RNA (GUCAGUGUGUUAAUCUUACAACCAGAACUCAAUUACCCCCUGCAUACACUAAUUCUUUCACACGUGGUGUUUAUU) template in molar ratio 1:1.5, in the same conditions.

To visualize oligonucleotides bound covalently to AbiA, 0.5 μM protein was incubated with 0.3 μM α-^33^P-dATP at 37°C, in the presence of reaction buffer described above for 1 min. Protein samples were analyzed on 12% dodecyl sulfate-polyacrylamide (SDS-PAGE) gel, scanned for radioactive signal and stained with Coomassie Brilliant Blue.

Fluorescently labeled RNA oligonucleotides for RNase activity of AbiA HEPN domain assays were synthesized by Future Synthesis. Four RNAs were tested: RNA1: FAM-AGAGAGUUUGAGAGAGAGAG, RNA2: ACUUAUCAGAUCCCUCGAGAAGCUGCGGGUACC-FAM, RNA3: GGUAGGGCCCACCCGGGAUCUUUGAUCCCGGGUGGGCUAUGUA-FAM (hairpin structure) and RNA4: AUAUUAUUUUAU-FAM. RNase assays were performed with a protein concentration of 1 μM and RNA substrates concentration of 1 μM, in a reaction buffer consisting of 50 mM Tris (pH 7.0), 250 mM NaCl, 10 mM MgCl_2_ and 5 mM dithiothreitol (DTT). The reactions were incubated at 37°C for 5, 30 and 60 min and then stopped by the addition of 40 mM EDTA and Proteinase K. Reaction products were then analyzed on 20% denaturing TBE-urea polyacrylamide gels and visualized by fluorescence readout with the Amersham Typhoon (Cytiva) biomolecular imager.

### Nanopore sequencing data processing

Raw data were basecalled with Dorado 0.5.1 (ONT) using the most accurate basecalling model (dna_r9.4.1_e8_sup@v3.3) and producing fastq as the output. Sequence lengths and nucleotide content for each sequencing read were calculated using seqtk (https://github.com/lh3/seqtk) function ‘comp’. Obtained data were loaded into R 4.2.1 where plots were produced using ggplot2 (37). As only the complementary DNA strand was sequenced, nucleotide frequencies were transformed to represent the original strand. Raw nanopore sequencing data are deposited at the European Nucleotide Archive; accession number PRJEB71919.

### Multi-angle light scattering

Molecular masses of AbiA variants were measured by size exclusion chromatography-multi-angle light scattering (SEC-MALS) using a system that consisted of an Akta Purifier liquid chromatography system (GE Healthcare) with an inline Dawn 8+ MALS detector (Wyatt) and Optilab T-rEX refractive index detector (Wyatt) on a Superdex 200 Increase 10/300 GL column (Cytiva). Samples (100 μl) were applied to the column that was equilibrated with a buffer that contained 25 mM Tris (pH 7.0), 400 mM NaCl, 2% glycerol and 1 mM DTT and resolved at 0.75 ml/min. Molecular masses were assessed with Astra 6 software (Wyatt) using the differential refractive index for real-time concentration measurement and a d*n*/d*c* value of 0.185 ml/g.

### Phage clustering based on proteome similarity

Phages from the BASEL collection as well as nine common laboratory phages (38) were clustered by proteome similarity. First, all proteomes were merged and clustered using MMseqs (39) with parameters *–cluster-mode 1 –cov-mode 0 -c 0.7 –min-seq-id 0.3*. Proteins that cluster together were considered as a set of homologues. Second, pairwise similarity scores (*sim(i,j)*) that reflect the number of shared homologous proteins between *i*th and *j*th phages normalized to the proteome size of the *i*th phage were computed for all phages. To do so, we used the following formula: *sim(i,j) = length(overlapped cluster)/length(proteome_i)*. Third, a symmetric proteome composition distance (PCD, *dist(i,j)*) matrix was then generated with values defined as *dist(i,j) = 1 – (average(sim(i,j), sim(j,i)))*. Next, the PCD matrix was used in hierarchical clustering in R with the average-linkage method resulting in 14 distinct phage clusters (Supplementary Table S1), and the resulting clustering was visualised using the C*omplexHeatmap* library (40, Supplementary Figure S1). All phages were annotated with family, subfamily and genus taxonomic levels from the NCBI Taxonomy database (41, January 2024 update) and ICTV (42, 2022 update). Similarity scores matrix, R script and associated data needed to reproduce the clustering and visualisation are available at figshare [dx.doi.org/10.6084/m9.figshare.24968247].

### Experimental phage infections

Efficiency of plaquing (EOP) assays were performed essentially as described previously using the BASEL collection and a set of common laboratory phages (30,38). Overnight cultures of *E. coli* BW25113 cells carrying either the empty vector [pJD1423; a pBR322 derivative (30) or the same plasmid with an Abi gene cloned under the P_tet_ promoter pJD1423-AbiA (VHp1642) or pJD1423-AbiK (VHp1639)], were mixed with top agar (LB with 0.5% agar, 20 mM MgSO_4_ and 5 mM CaCl_2_), to a final concentration of 0.075 OD_600_ units/ml, and 10 ml overlayed on square (12 cm × 12 cm) LB-agar plates (1.5% agar). Phage stocks were 10-fold serially diluted in SM buffer (0.1 M NaCl, 10 mM MgSO_4_, and 0.05 M Tris–HCl pH 7.5) and 2.5 μl of each of eight dilutions spotted on solidified top agar plates. Plaque formation was monitored after 6 and 24 h of incubation at 37°C. Phages that showed sensitivity to expression of either AbiA or AbiK were re-tested towards both systems using three different transformants of each plasmid. Plaques were counted after 6 h at 37°C and the EOP calculated for each repeat by dividing the plaque forming units for the Abi system by that for the empty vector. The average EOP value from the three repeats were –log_10_-transformed and used to generate the heat map (log_10_ protection).

For phage infection of liquid cultures, three different transformants of *E. coli* BW25113 carrying either the empty vector or an Abi-expressing plasmid were grown overnight in LB medium containing 100 μg/ml ampicillin, 10 mM MgSO_4_ and 2.5 mM CaCl_2_. The cells were diluted to OD_600_ ≈ 0.075 in the same medium and 100 μl were added to wells of a 96-well plate. Freshly prepared stocks of relevant phages were diluted in SM buffer to generate final MOI values of 10, 1 and 0.1 when 10 μl of the dilution was added to a well. Ten μl of SM buffer were added to control wells. Bacterial growth was monitored at 37°C in a Synergy H1 (BioTek) plate reader by measuring OD_600_ every 15 min.

## Results

### AbiA structure determination

The first structures of polymerases that are related to RTs and involved in abortive infection were recently reported for the AbiK and Abi-P2 subfamily representatives (25). However, no structural or mechanistic information has been available for the third group of Abi RTs, AbiA. These enzymes are distantly related to AbiK and Abi-P2, and their unique feature among Abi polymerases is the presence of a C-terminal HEPN domain. HEPN domains are often found in various proteins that are involved in antiviral mechanisms. Our aim was to determine the structure of AbiA and its biochemical properties. We chose the well-studied plasmid-encoded protein that was originally isolated from *L. lactis* (20,21,43). Recombinant AbiA was overexpressed in *E. coli* and purified. Despite nuclease treatment during purification, the protein sample contained nucleic acids, as demonstrated by the absorbance ratio at wavelengths of 260 and 280 nm, which exceeded 1.0. This was similar to AbiK polymerase, which was previously shown to co-purify with single-stranded DNA fragments that were covalently bound to its priming tyrosine (25). We assumed that the nucleic acid that was present in the AbiA sample was also covalently attached to the protein.

In the crystallization trials, we initially used AbiA without a tag. This protein did not crystallize, so we used His_6_-SUMO-AbiA fusion protein, which readily produced crystals under a broad range of conditions. We obtained AbiA crystals that diffracted X-rays to 2.8 Å resolution. The crystal structure was solved by molecular replacement using a model that was generated with AlphaFold2 (34) as the search model. The structure was refined to good geometry and statistics, with an *R*_free_ of 26% (Supplementary Table S2, Supplementary Figure S2).

### AbiA monomer consists of three domains

**Figure 1.**
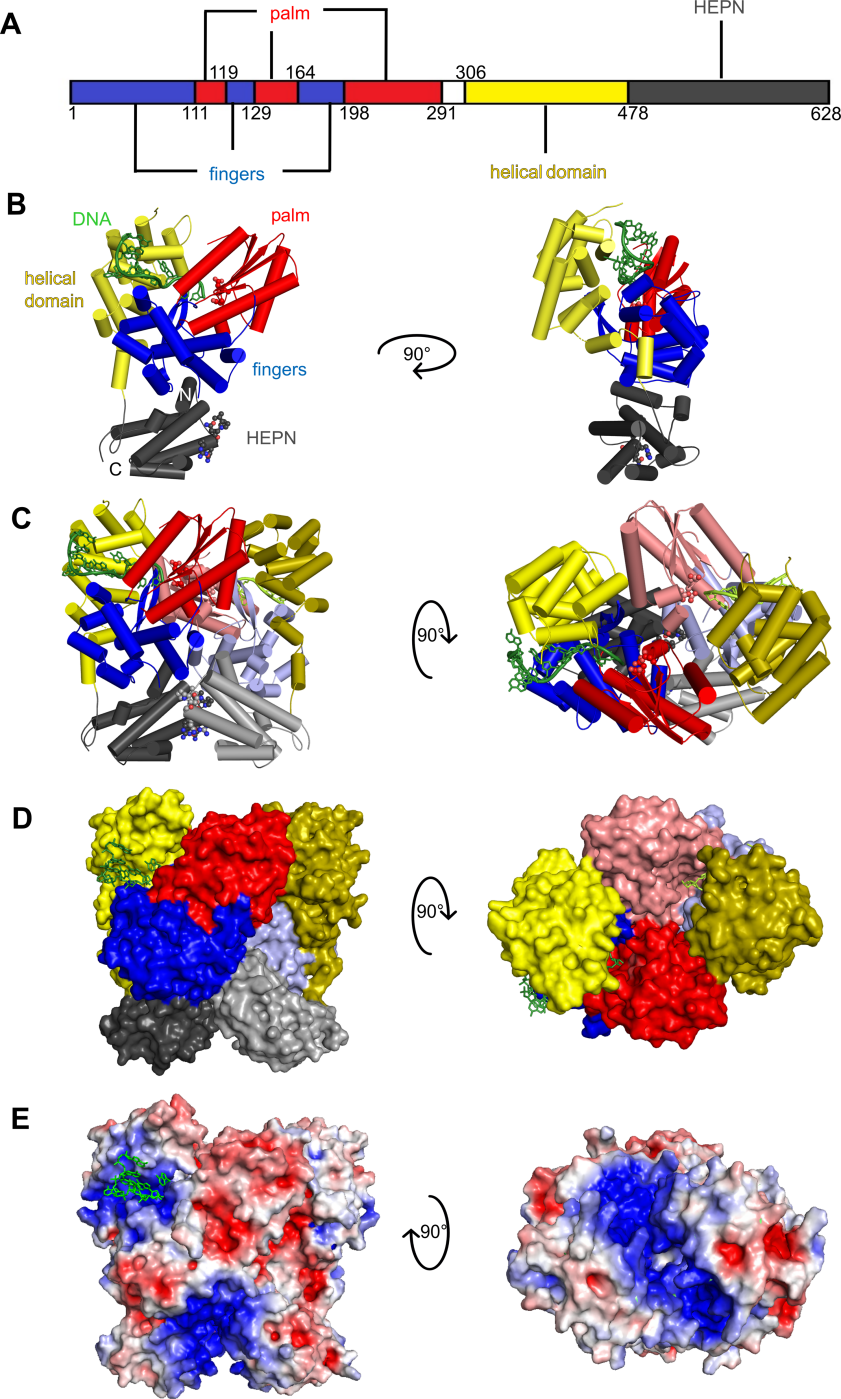
Overall structure of AbiA-DNA complex. (**A**) Domain organization of AbiA protein. Numbers indicate residues at the domain boundaries. Protein (sub)domains are color-coded (fingers: blue; palm: red; helical domain: yellow; HEPN: dark gray). The flexible loop connecting polymerase domain and helical domain is shown in white. (**B**) Crystal structure of AbiA monomer in cartoon representation, shown in two orientations obtained by 90 degree rotation around the y-axis. Protein (sub)domains are color-coded as in (A). DNA is shown as green sticks. Conserved aspartic acid residues within the polymerase active site are shown as sticks and spheres. Residues within HEPN active site are shown as sticks and spheres. (**C**) Crystal structure of AbiA dimer shown in two orientations obtained by 90° rotation around the x-axis. Two subunits of the dimer are shown in cartoon representation. Subdomains of one subunit are colored as in (A) and for the other subunit the subdomains are colored: fingers: light blue, palm: pink, helical domain: olive, HEPN domain: light gray. (**D**) Crystal structure of AbiA dimer shown in surface representation, with color coding as in (C). (**E**) Electrostatic potential of AbiA dimer surface; the potential scale is rendered from the -5 kTe^−1^ to +5 kTe^−1^ from red to blue. Please note that in (E) the rotation between panels is in a different direction than in (C, D).

The AbiA structure consists of three distinct domains: an N-terminal RT-like polymerase domain, a helical domain (which is unique to Abi polymerases), and a C-terminal HEPN domain (Figure 1A, B). The RT-like domain in AbiA contains palm and fingers subdomains that are found in all RTs but lacks the thumb subdomain that is present in canonical RT structures. The thumb subdomain in other polymerases is responsible for recognition and positioning of the nascent double-stranded nucleic acid. Similar to AbiK and Abi-P2 (25), the expected position of the thumb subdomain in AbiA is occupied by the N-terminal portion of the helical domain. This architecture is likely an adaptation to the type of nucleic acid that is synthesized by Abi polymerases, which is single-stranded.

The AbiA palm subdomain contains four α-helices and four β-strands, forming a small β-sheet structure that comprises the RYVDD motif. The two conserved aspartic acid residues in this motif form the polymerase active site. In the fingers subdomain there are nine α-helices and a hairpin structure that is composed of two β-strands. The helical domain consists of 12 α-helices. They form an α-solenoid with a curved, bilobal shape. The HEPN domain is exclusively composed of α-helices. It is located in the C-terminal region of the protein and separated from the helical domain by an unstructured loop of 25 amino acids (Figure 1B).

In the structure, we observed additional electron densities corresponding fragments of ssDNA. In different protein chains they comprised 5–9 nt. Untemplated DNA synthesis by Abi polymerases is known to result in random sequences (24). Therefore, in our structure, we built DNAs with arbitrarily chosen sequences.

### AbiA dimer formation is mediated by the HEPN domain

In the crystal of AbiA, the asymmetric unit contains four molecules of the protein–DNA complex, forming a dimer of dimers (Supplementary Figure S3). The two dimers in the asymmetric unit are nearly identical, with a calculated root mean square deviation (RMSD) of the position of 1168 C-α atom pairs equal to 1.1 Å. Within a dimer, the RMSD of the position of 592 C-α atom pairs is equal to 1.0 Å. The main interaction interfaces within the AbiA dimer are formed between long helices from HEPN domains (residues 510–540 and 565–585) of each protomer (Figure 1C–E). Another region that is involved in dimer formation is a long α-helix from the palm subdomain, comprising residues 251–266, which contacts the first two helices from the helical domain and the unstructured loop between them from the neighboring chain (region that comprises residues 315–340). The analysis of intersubunit contacts with PDBePISA (44) indicated that the average interface area between two protein chains within a dimer is 2172 Å^2^. According to PDBePISA calculations, AbiA dimer—but not tetramer—should be stable in solution.

**Figure 2.**
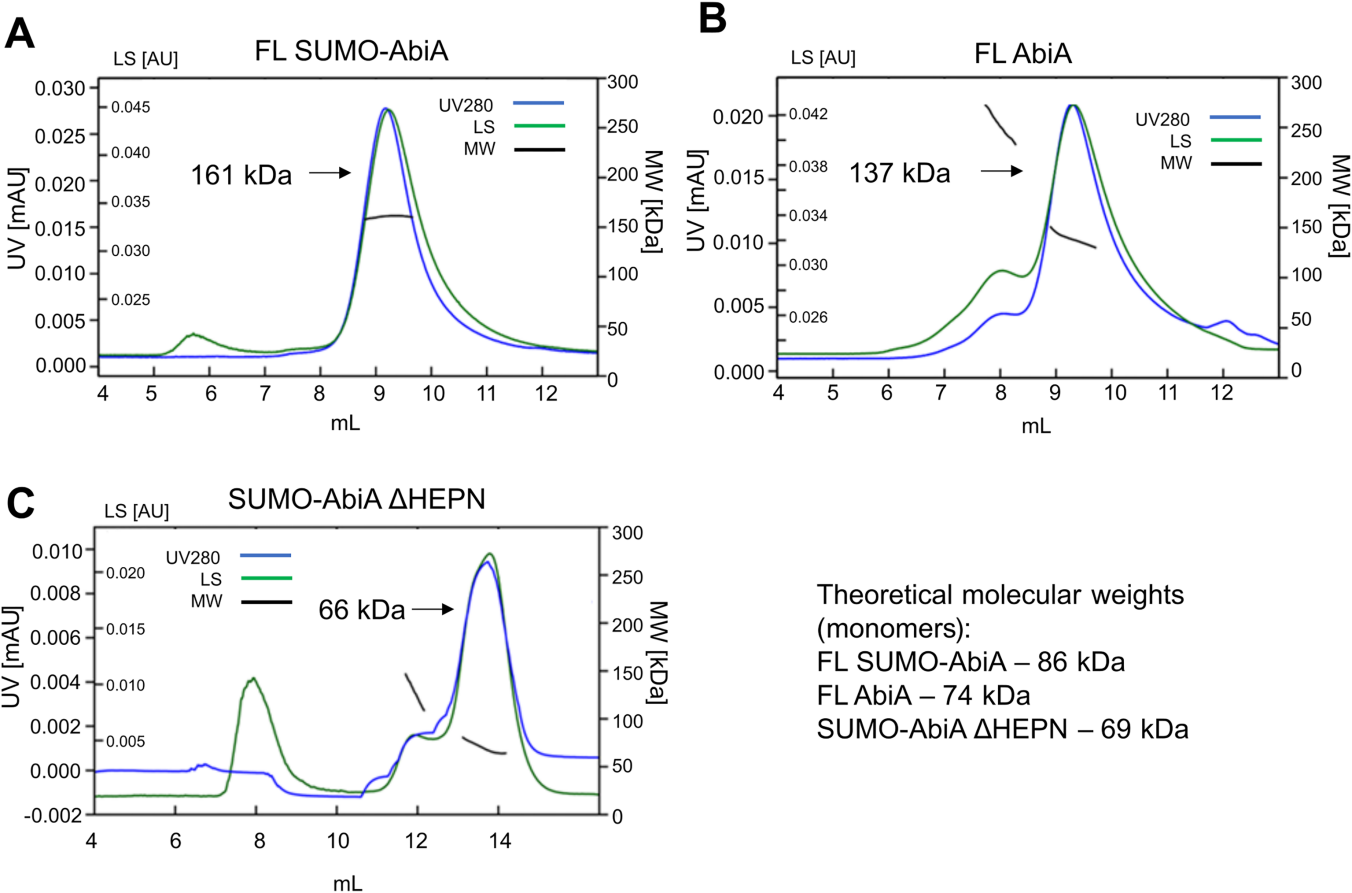
Gel filtration/multi-angle light scattering analysis of AbiA variants. (A–C) Elution traces from a Superdex 200 Increase 10/300 GL column for (**A**) full-length SUMO-tagged AbiA, (**B**) full-length untagged AbiA, and (**C**) SUMO-tagged AbiA truncated variant that lacked the HEPN domain. Absorbance at 280 nm is shown in blue (UV280), and light scattering at 90° is shown in green (LS). The estimated molecular weight (MW) values are shown in black. The values of molecular weight estimates for the main peaks at their apex (Mp) are indicated with arrows.

To confirm the oligomeric state of AbiA in solution, we performed gel filtration/MALS experiments (Figure 2). SUMO-AbiA eluted as a peak with a molecular weight of 161 kDa (Figure 2A; theoretical molecular weight of a dimer equals 171 kDa). As SUMO proteins themselves can form multimers (45), we also performed this experiment using untagged AbiA. Untagged AbiA eluted as a dominant peak with a molecular weight of 137 kDa (Figure 2B; theoretical molecular weight of a dimer equals 148 kDa), further corroborating its dimeric nature in solution.

The presence of a C-terminal HEPN domain is a distinguishing feature of the AbiA subfamily among Abi polymerases (46). In bacteria, HEPN domains are common in toxic effectors of toxin-antitoxin (TA) systems (47), effector proteins from diverse anti-MGE defense systems such as restriction-modification (R-M) systems (48), and in the C-terminus of Csm6 and Csx1 proteins implicated in CRISPR antiviral defense (29). Apart from AbiA, HEPN is present in AbiV, AbiF, AbiD, and AbiJ abortive infection systems (29). HEPN domains are often associated with nucleolytic activity or RNA binding (48). Most catalytically-competent HEPN domains possess a motif with arginine and histidine residues that are separated by four or six other amino acids (R-X_(4–6)_-H) (48). This motif is highly conserved between HEPN domains in different organisms, despite low identity of the overall sequences. The presence of this motif correlates with the nucleolytic activity of proteins that possess HEPN domains, and nucleases with a HEPN domain have been shown to lose their activity upon substitution of the histidine in the R-X_(4–6)_-H motif (48–50). Proteins in the HEPN family that lack this motif are considered mostly non-catalytic RNA-binding domains, such as in the case of the MtlR mannitol repressor (48). In the *L. lactis* AbiA HEPN domain, the RNSNPVSH motif (residues 581–588) contains six residues between arginine and histidine. The presence of this motif indicates that the HEPN domain of AbiA may have nucleolytic activity. To test this, AbiA was incubated in a reaction buffer with several different single-stranded RNA substrates, labeled with fluorescein at either 3′ or 5′ end, with lengths ranging from 12 to 43 nt; one of them formed a hairpin structure with a double-stranded region (see Materials and methods for sequences). In this experiment, we did not observe any RNA degradation activity (Supplementary Figure S4). Thus, we concluded that the HEPN domain likely plays a structural role. We also noted that HEPN domains in the AbiA dimer form a positively charged cleft that may serve for nucleic acid binding (Figure 1E). Further studies will be required to test whether this region binds RNA or DNA and what are the functional consequences of this binding.

The AbiA structure that is described herein indicates that the AbiA HEPN domain can also participate in AbiA dimer formation. To confirm whether the HEPN domain is responsible for AbiA dimer formation, we performed MALS experiment with a truncated AbiA-ΔHEPN (aa 1–478) construct, in which the HEPN domain was removed. Indeed, the elution profile of truncated, SUMO-tagged AbiA-ΔHEPN contained a predominant peak with the molecular weight of 66 kDa (theoretical molecular weight of a monomer is 69 kDa), consistent with previous reports of the HEPN domain playing the key role in protein dimerization (29,51).

### Structural and sequence comparison of AbiA with other Abi and non-Abi RTs

**Figure 3.**
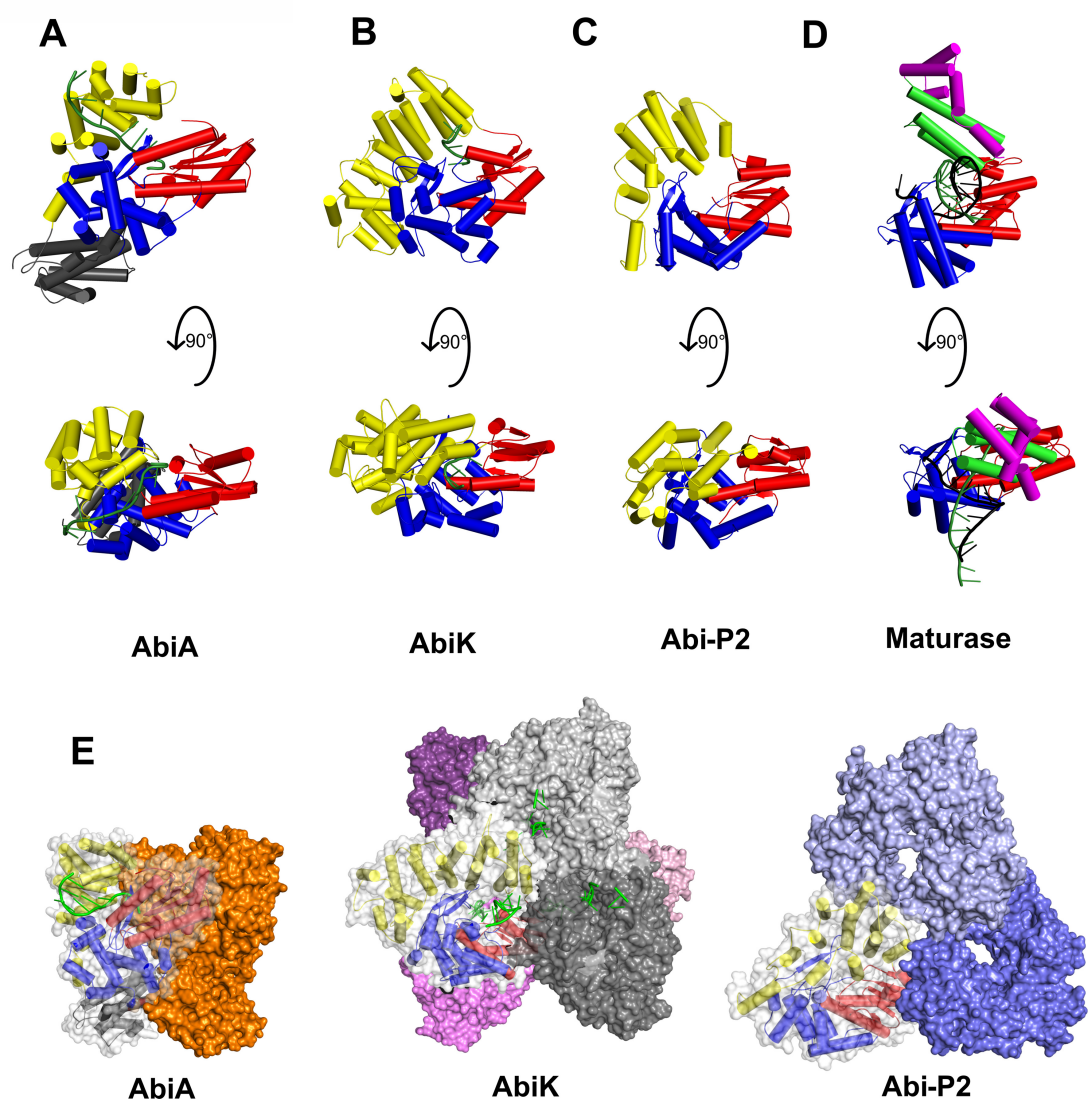
Comparison of structures of Abi polymerases and group II intron maturase. The structures of (**A**) AbiA (this work), (**B**) AbiK (PDB ID: 7R07), (**C**) Abi-P2 (PDB ID: 7R08) and (**D**) group II intron maturase (PDB ID: 6AR1) are shown in two orientations obtained by 90 degree rotation around the x-axis. The colors correspond to the respective (sub)domains: red (palm subdomain), blue (fingers subdomain), yellow (helical domain), green (thumb subdomain), magenta (DNA-binding domain), and gray (HEPN domain). The ssDNA in the AbiA and AbiK structures is shown in green, and the double-stranded hybrid in the maturase structure is shown in black for RNA and green for DNA. (**E**) Comparison of the oligomeric state of Abi RTs: AbiA dimer (left), AbiK hexamer (middle) and Abi-P2 trimer (right).

Despite sequence divergence, the AbiA RT-like polymerase domain exhibits significant similarities not only to known Abi-P2 and AbiK structures (Figure 3A–C) but also to distantly related RTs, such as group II intron maturases, especially within the palm subdomain (Figure 3D). With nine α-helices, the AbiA fingers subdomain is significantly larger than the fingers subdomain of maturase but comparable to fingers subdomains of Abi-P2 and AbiK. Nine conserved RT motifs within the palm and fingers subdomains of group II intron polymerase have been identified (52). Three of these (motifs 3, 5 and 6) can be unambiguously aligned between AbiA and other RTs. Within these motifs, AbiA displays 25.3% sequence identity with Abi-P2 and 27.9% sequence identity with AbiK. Structural similarity between Abi polymerases is moderate (Supplementary Figure S5A). Between Abi-P2 and AbiA RT domains, the RMSD value for 216 Cα pairs is 4.9 Å. For AbiK-AbiA superposition, the corresponding value is 4.8 Å for 240 Cα atom pairs (Figure 3A-C). The strongest conservation is observed for the RT-like core, whereas helical domains of Abi polymerases are similar only in overall shape and not in sequence (Supplementary Figure S5B). The HEPN domain from AbiA has no counterpart in other Abi polymerases. A DALI search for structures that are similar to the AbiA HEPN domain indicated moderate (*Z* factor = 8.0–9.0) similarity to human sacsin and HEPN domains from toxin–antitoxin systems, among others (53,54, Supplementary Figure S5C).

From the structural perspective the most important difference between Abi RTs from the three clades – AbiK, Abi-P2 and AbiA – is the oligomerization state. AbiK has been shown to form hexamers (dimer of trimers) and Abi-P2 was found to be a trimer (25). In contrast AbiA is a dimeric protein (Figures 1C–E, 3E).

### Protein priming and residues involved in AbiA activity

**Figure 4.**
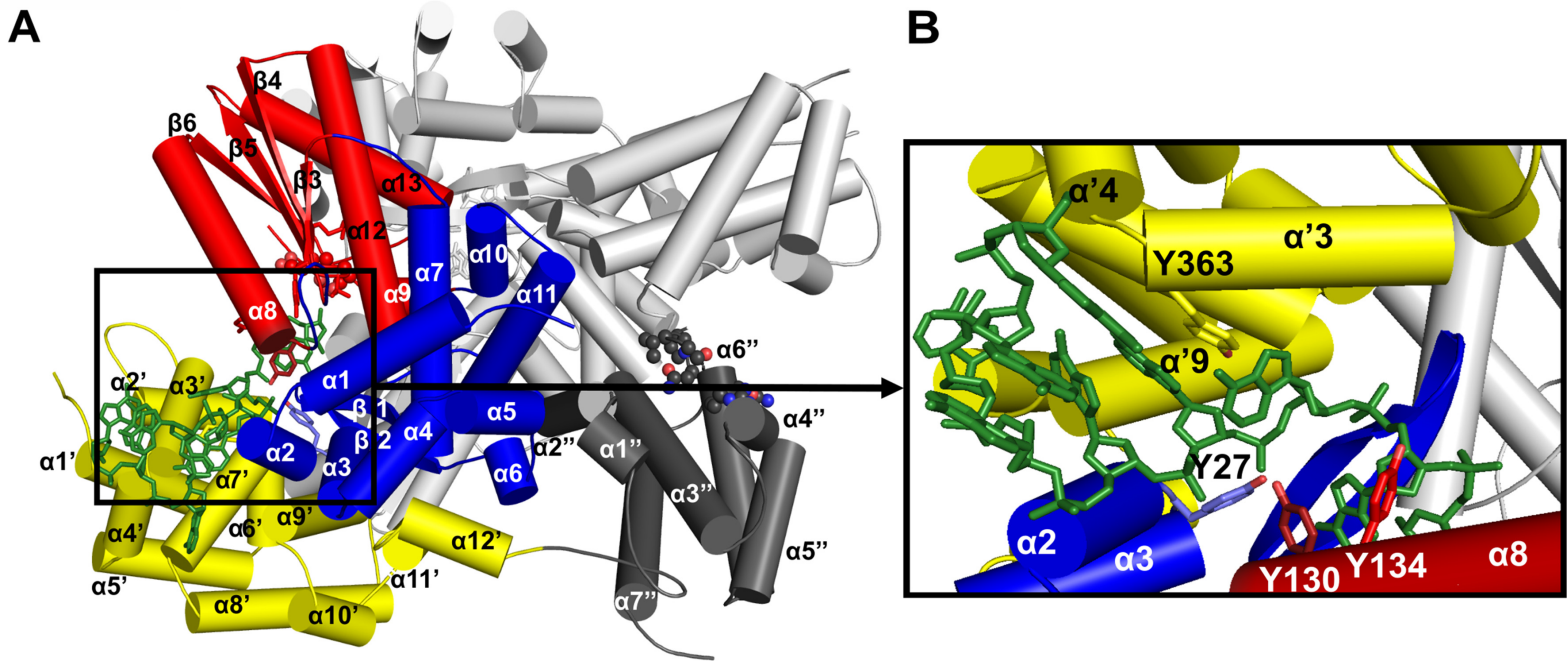
Protein–DNA contacts in AbiA–DNA complex structure. (**A**) AbiA monomer with bound ssDNA product, colored as in Figure 1A. The palm subdomain of the polymerase domain is shown in red. The fingers subdomain is shown in blue. The helical domain is shown in yellow. The HEPN domain is shown in gray. DNA is shown in green. The helices and β-strands are numbered, those that belong to the helical domain are marked with prime, and those that belong to the HEPN domain are marked with double prime. Position of the second monomer is indicated in light gray. (**B**) Amino acid residues that stabilize the nascent DNA strand. DNA is shown as sticks. Amino acid residues that are involved in contacts with the DNA are shown as sticks and labeled.

The Abs_260_/Abs_280_ ratio of the purified AbiA sample exceeded 1, indicating the presence of a nucleic acid despite nuclease treatment during purification. Abi polymerases are known to perform protein-primed, untemplated DNA synthesis (24,25). Therefore, we expected that the nucleic acid that co-purified with the protein was covalently attached to a specific priming residue. In the crystal structure of AbiA, short (5–9 nt) single strands of DNA could be observed for each of the four protein chains (Figure 4A). DNA was most likely synthesized in the bacterial cell and co-purified with the protein. The AbiA-DNA complex structure allowed us to identify residues that stabilize nascent DNA. Tyr130, Tyr134 and Tyr363 interact with the DNA strand through stacking interactions between aromatic rings of the side chains and nucleobases. Another residue that is involved in DNA contacts is Tyr27, which interacts with the phosphate backbone (Figure 4B). The tyrosine residues that interact with the DNA belong to different AbiA (sub)domains: fingers, palm and helical domain.

**Figure 5.**
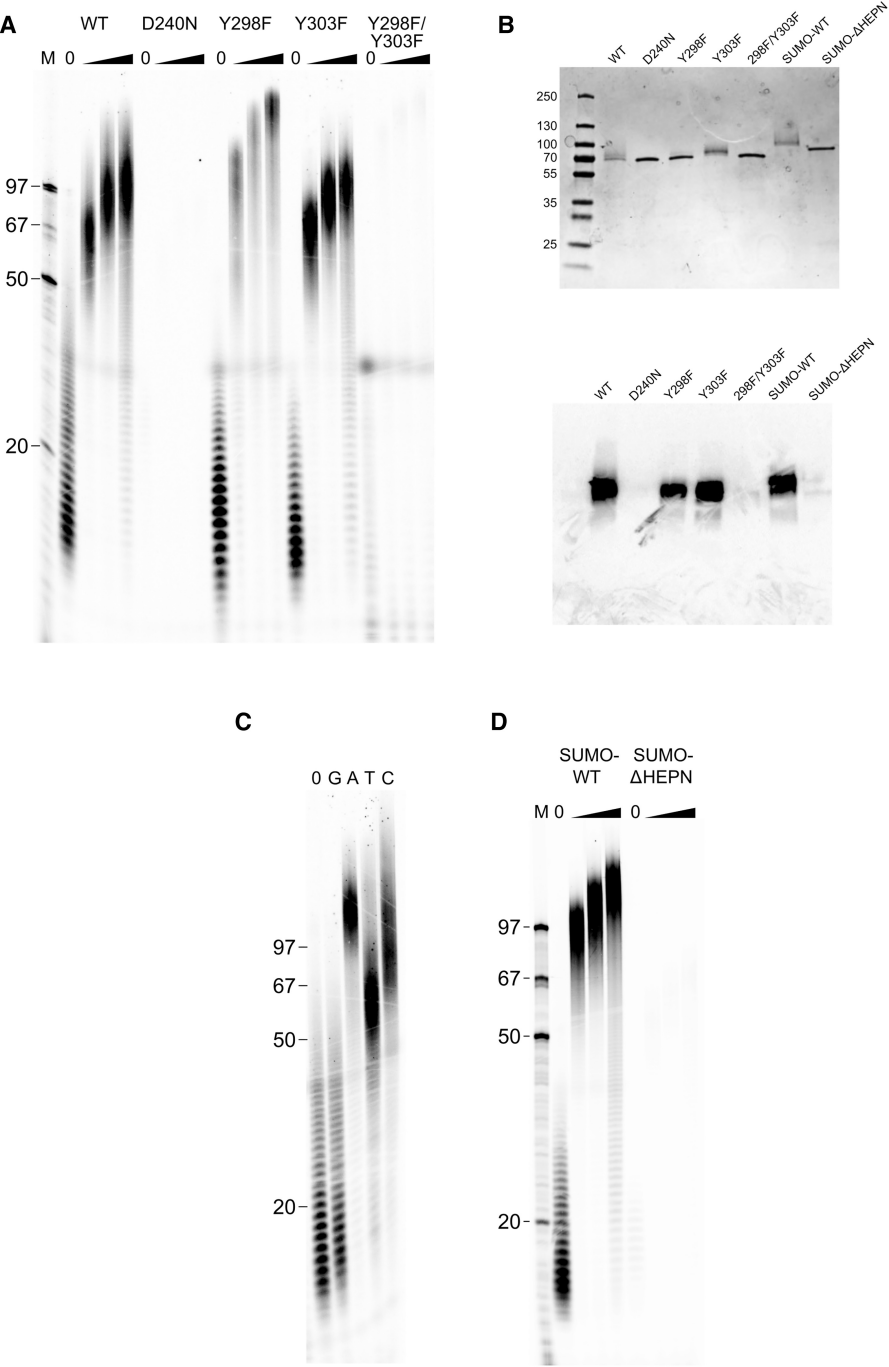
DNA polymerization assay for AbiA wild type protein and its variants (radioactivity readout). (**A**) Template-independent polymerization activity of AbiA variants. Protein (0.5 μM) was incubated with 5 μCi α-^33^P-dATP (concentration 0.16 μM) at 37°C for 1 minute in the reaction buffer (50 mM Tris [pH 7.0], 250 mM NaCl, 10 mM MgCl_2_, and 5 mM DTT), to label primers copurified with protein. After labelling, half of the reaction was diluted fourfold in the reaction buffer containing an equimolar mix of 150 μM of all four deoxynucleotide triphosphates and aliquots of primer extension reactions were collected to a stop mix containing EDTA and proteinase K. Reaction products were analyzed on 15% TBE-urea polyacrylamide gels and visualized by autoradiography. Each set of four reactions consists of a primer labelling reaction (diluted four-fold in water, lane ‘0’) and primer extension time points (1.5, 3 and 4.5 min—black triangle). M – Single-stranded DNA marker made of 5′end-labeled oligonucleotides. (**B**) Analysis of labeled nucleotides covalently attached to AbiA variants. Protein (0.5 μM) was incubated with α-^33^P-dATP (0.16 μM) at 37°C in the presence of reaction buffer for 30 min. Protein samples were analyzed on a 12% SDS-PAGE gel and visualized by autoradiography (lower panel), then stained with Coomassie Blue (upper panel). M, protein size marker. (**C**) Deoxynucleotide preference of template-independent polymerization activity of AbiA. Aliquots of primer labelling reaction of wt AbiA (prepared as described in A) were diluted twofold in four extension mixes containing the reaction buffer with 200 μM of dGTP, dATP, dTTP or dCTP. Primer extensions were stopped after 5 min. Reaction products were analyzed on 15% TBE-urea polyacrylamide gels and visualized by autoradiography. M – Single-stranded DNA marker made of 5′end-labeled oligonucleotides. (**D**) Template-independent polymerization activity of SUMO-AbiA variants. For description see (A).

We could not observe a DNA electron density for the initial nucleotides that were attached to the protein. Thus, the priming residue could not be identified directly based on the structural data. Protein-primed template-independent DNA polymerase activity was previously hypothesized for AbiA (55), but has not been demonstrated in biochemical assays. Therefore, to test AbiA for this activity we established a polymerase activity assay, which was based on addition of α-^33^P-dATP to label the DNA that co-purified with AbiA, followed by extension of this DNA in a mix containing equimolar dNTPs concentrations (Figure 5). In the presence of α-^33^ P-dATP only, we observed radioactive products of 10–40 nt in length with maximum intensity around 18 nt (the lengths are calculated with subtracting one nucleotide incorporated during labeling, Figure 5A). Addition of a dNTP mix to the reaction resulted in extension of the labeled primers and formation of a heterogenous pool of products with an average size around 100 nt. We used His-tagged AbiA proteins without SUMO tag (wild type and substitution variants) and the reaction buffer was optimized by varying pH and salt concentration to obtain the highest polymerase activity. The reactions did not contain any additional nucleic acids that could serve as primers, but we note that the crystal structure showed the presence of pre-synthesized ssDNA fragments that could be extended by the addition of nucleotides.

The reaction with α-^33^P-dATP was performed with ∼3-fold molar excess of protein. Under these single-turnover kinetic conditions incorporation of hot ATP is expected to result in radioactive labelling of DNA which co-purified with AbiA instead of polymerization of α-^33^P-labeled poly-dA DNA chains. We observed radioactive products of 10–40 nt in length with maximum intensity around 18 nt (the lengths are calculated with subtracting one nucleotide incorporated during labelling, Figure 5A). As a negative control in our experiments, we used the D240N variant, in which a conserved active site residue of the RT-like domain was substituted. This variant did not show any labeling of the DNA. Based on the results with the wild type protein we concluded that 10–40 nt is the distribution of the lengths of the DNAs which co-purified with AbiA. We used this information to assess the fraction of AbiA molecules which contain attached DNA. The weighted average length of the covalently attached ssDNA strand (18.3 bases) was estimated based on densitometric quantitation of products of the labeling reaction. Next, we calculated a theoretical absorbance value at 260 and 280 nm wavelength assuming that each protein molecule is covalently attached to a ssDNA fragment. We used the experimentally obtained absorbance values for a protein variant without DNA (D240N) and a calculated absorbance value for a 18.3-base-long ssDNA. By comparing the Abs_260_/Abs_280_ ratio measured for wild type AbiA to the value measured for AbiA without any DNA (D240N) and a theoretical value calculated for the 100% homogeneous AbiA-DNA adduct, we estimated the DNA occupancy to be above 90% in the wild type AbiA sample. This also indicates that in most of AbiA dimers both subunits carry the ssDNA products.

We assumed that the DNA which co-purified with AbiA was covalently attached to the protein. To verify this, we resolved the samples after the labeling reaction on a denaturing SDS-PAGE, visualized the gel by autoradiography and stained it with Coomassie Brilliant Blue (Figure 5B). The radioactive signal was observed for the wild type protein, indicating the formation of a covalent protein-DNA adduct (Figure 5B). D240N variant did not show any radioactive signal which indicated a lack of covalently attached DNA.

Based on AbiA crystal structure we predicted that the residue responsible for protein priming could be located in the 290–310 loop, which connects the RT domain and the helical domain of the protein. This loop was partly disordered in our structure due to its flexibility. Analysis of the structure also indicated that this flexible region could reach into the active site for the attachment of the first nucleotide of the DNA. There are two tyrosine residues in 290–310 region to which the DNA could be covalently linked: Tyr298 and Tyr303. To test the role of these residues in protein priming we prepared single substitution variants of AbiA – Y298F and Y303F – as well as a double substitution variant Y298F/Y303F. Y298F showed similar activity to wild type protein and it generated longer products in the polymerase assay (Figure 5A). The activity of Y303F was even higher than wild type AbiA activity but it generated shorter products than Y298F. Importantly, the double-substituted variant Y298F/Y303F was devoid of polymerase activity. The products of the labeling reaction were also analyzed by SDS-PAGE (Figure 5B). For Y298F and Y303F variants both proteins had radioactive signal showing covalent attachment of ^33^P-dAMP. In contrast, the Y298F/Y303F variant failed to produce this signal. These results showed that both Tyr298 and Tyr303 can be used for protein priming with each residue leading to different polymerization efficiencies. Removal of hydroxyl groups of both these residues blocked the DNA polymerization.

To check whether AbiA has the ability to extend a non-covalently bound primer, we took advantage of the fact that AbiA Y298F/Y303F has an intact polymerase active site but does not contain covalently attached DNA which could occlude the DNA-binding cleft. We mixed AbiA Y298F/Y303F with fluorescently labeled 18 nt DNA either alone or hybridized to a 75-nt RNA or DNA template whose 3′ terminal region was complementary to the primer. We performed a polymerization reaction in the presence of a mix of dNTPs. We did not observe any reaction products (Supplementary Figure S6), so we conclude that, at least under the conditions used in our assay, AbiA requires covalent attachment of the DNA for the polymerization to occur.

**Figure 6.**
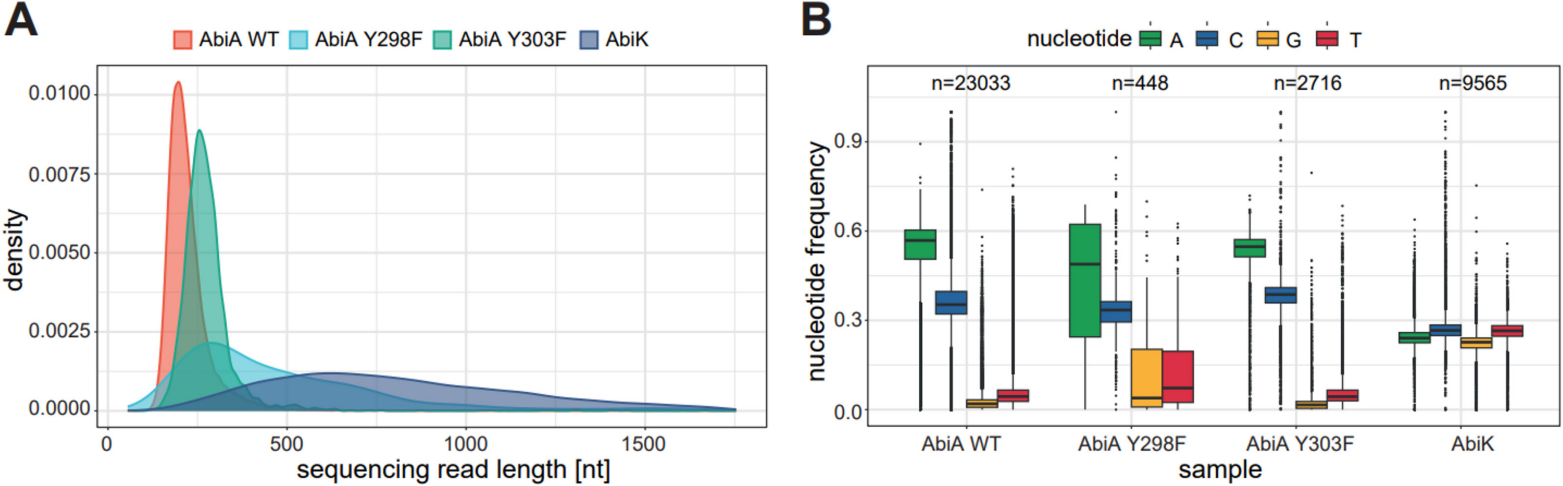
Nanopore sequencing of AbiA polymerization products. (**A**) Distribution of read lengths for each library sequenced. (**B**) Nucleotide frequency for original AbiA-synthesized DNA strands. The number of sequencing reads included in the analysis is indicated for each sample.

We also wanted to verify the nucleotide preference of AbiA. We first labeled DNAs co-purifying with AbiA using α-^33^P-dATP as described above. We then incubated the mixture with single dNTPs (Figure 5C). dATP and dCTP were incorporated with efficiencies similar to those observed with dNTP mix. dTTP was incorporated less efficiently producing shorter products. Addition of dGTP did not lead to the primer extension. To study the nucleotide preference further we sequenced DNA products generated by wild type, Y298F and Y303F AbiA using Nanopore platform. ssDNA generated by the AbiK protein was used as a control. The complementary DNA strand was synthesized and the resultant dsDNA was subjected to adaptor ligation followed by Nanopore sequencing. As the covalently attached tyrosine residue remained attached to the 5′ end of the original DNA strand after AbiA degradation by proteinase K, preventing the adaptor ligation, only the complementary strand was sequenced, allowing a strand-specific analysis. Obtained read lengths were generally in agreement with the *in vitro* polymerization assay (Figure 6A). Nucleotide content analysis revealed that AbiA-synthesized fragments contained almost exclusively adenosines and cytosines (Figure 6B), confirming, in agreement with the *in vitro* activity assay (Figure 5C), enzyme's preference towards these nucleotides. Incorporation frequencies were similar for all of the analyzed AbiA variants. At the same time, AbiK-synthesized fragments had almost equal frequency of incorporation of all four nucleotides. Therefore, we conclude that AbiA, in contrast to AbiK, has a preference for adenosines and cytosines.

Finally, we sought to verify the importance of the HEPN domain and dimerization for AbiA activity. We tested the activity of the truncated, monomeric ΔHEPN variant. For this variant only, the SUMO fusion protein could be purified as His-tagged protein was unstable. Therefore, we used the SUMO fusion in activity assays, compared it to wild type SUMO-AbiA and found that the HEPN domain deletion made the protein inactive (Figure 5D). The ΔHEPN variant does not dimerize (Figure 2C), so the lack of enzymatic activity of this variant may stem from its inability to form dimers. We cannot exclude a possibility that the removal of HEPN domain also introduced other effects. We tried to study the effect of dimerization separately, but we were unsuccessful in generating point substitution variants that would be monomeric in solution.

### *Lactococcus* AbiA and AbiK provide antiphage defense in an *E. coli* surrogate host

We sought to validate our *in vitro* structure-functional insights through experimental phage infection assays. As phage defense systems commonly retain functional activity when studied in convenient surrogate hosts, we tested the antiphage activity of AbiA in *E. coli* challenged by diverse phages from the BASEL collection as well as a set of common laboratory phages (38). In parallel we also tested the other well-studied *Lactococcus* Abi RT–AbiK. Both systems were expressed in the *E. coli* K-12 strain BW25113 under control of the constitutive P_tet_ promoter from a pBR322-derived vector. Importantly, the growth of *E. coli* BW25113 was not affected by constitutive P_tet_-driven expression of AbiA or AbiK (Supplementary Figure S7).

**Figure 7.**
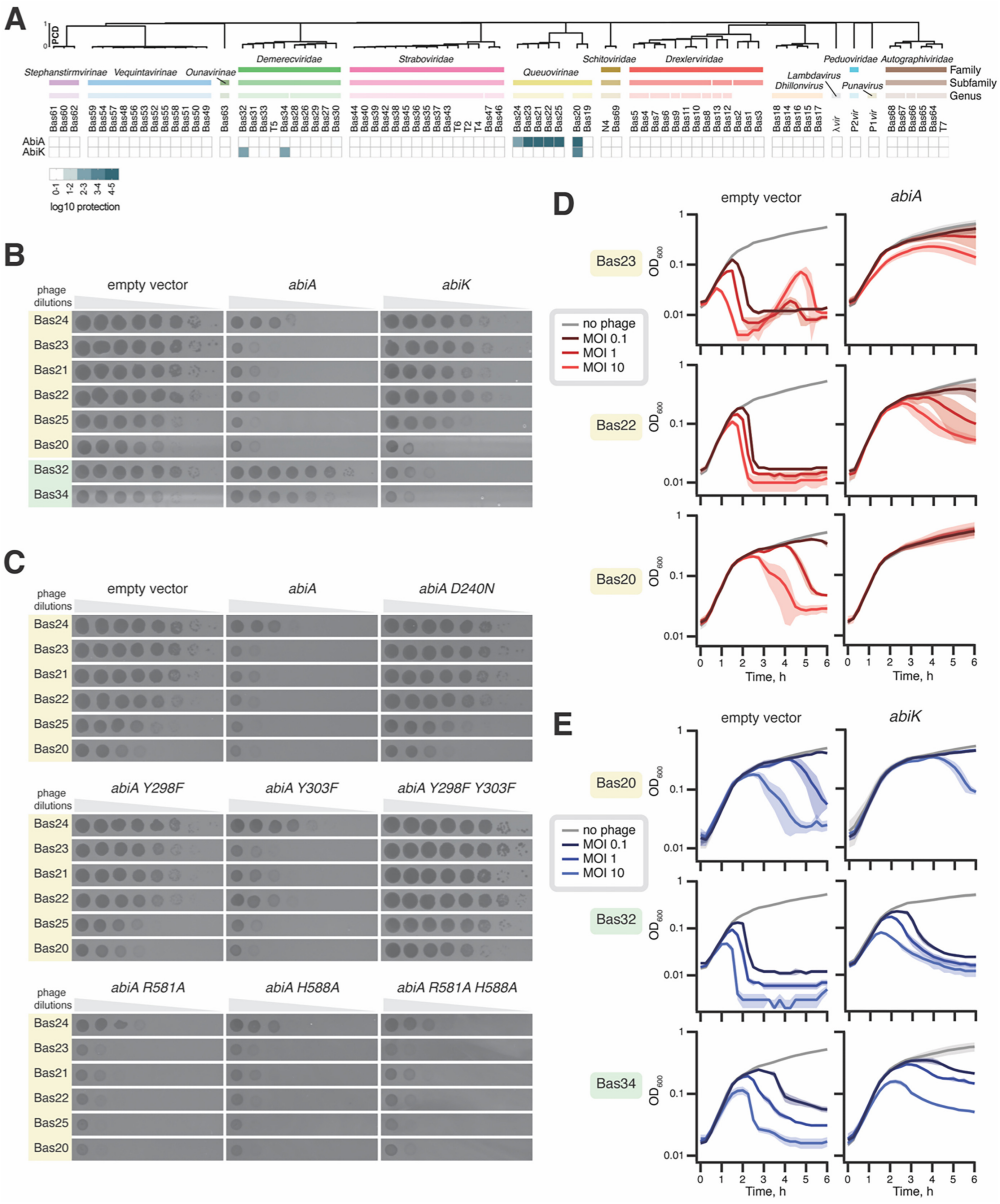
The AbiA and AbiK systems protect *E. coli* from distinct coliphages. (**A**) Defense profiles of AbiA- and AbiK-expressing *E. coli*. Top agar lawns of *E. coli* BW25113 harboring the empty pBR322-derived vector pJD1423 or the same plasmid expressing AbiA (VHp1642) or AbiK (VHp1639) were challenged with 10-fold serial dilutions of indicated phages (Supplementary Figure S8). The phages that showed sensitivity to the expression of either system were re-tested towards both systems using three different transformants for each plasmid. The average EOP value from the three repeats were -log_10_ transformed, generating the log_10_ protection value. The phage order and dendrogram are defined by hierarchical clustering applied to the Proteome Composition Distance (PCD) matrix (see Materials and Methods section and Supplementary Figure S1 for details). (**B**) Plaque assays on lawns of *E. coli* BW25113 harboring the empty vector (pJD1423) or the *abiA-* and *abiK*-containing plasmids (VHp1642 or VHp1639). The indicated phages were 10-fold serially diluted and spotted on top agar plates (**C**) Plaque assays on lawns of *E. coli* BW25113 carrying the empty vector (pJD1423) or the same plasmid expressing the indicated *abiA* alleles from the P_tet_ promoter (VHp1642, VHp1661-VHp1667). The indicated phages were 10-fold serially diluted and spotted on top agar plates (**D** and **E**). Growth of *E. coli* BW25113 harboring the empty vector (pJD1423) or a plasmid-encoded *abi* gene (VHp1642 or VHp1639) in the presence of indicated phages at MOIs of 0, 0.1, 1 and 10. The curves represent the average of three replicates, using different transformants in each replicate, and the shaded areas indicate the standard deviation.

Efficiency of plaquing (EOP) assays on bacterial lawns growing on top agar plates revealed that AbiA provided potent protection against the *Queuovirinae* phages Bas20-Bas25 (Figure 7A, B, Supplementary Figure S8). Out of seven tested phages in the *Queuovirinae* subfamily, AbiA failed to protect from only one, Bas19. AbiK granted protection against two out of ten tested phages in the *Demerecviridae* family: Bas32 and Bas34. In addition, AbiK protected against one phage in the *Queuovirinae* subfamily: Bas20. Thus, AbiA and AbiK provided protection against distinct but partially overlapping subsets of BASEL coliphages (Figure 7B, Supplementary Figure S8).

### Antiphage defense by AbiA requires the DNA polymerase activity

To probe the functional importance of the key residues in the RT-like and HEPN domains of AbiA, we performed plaque assays with the *Queuovirinae* phages that AbiA is active against. The D240N polymerase active site AbiA variant was, as expected, inactive in antiphage defense. In agreement with our *in vitro* polymerase activity assays, the Y298F/Y303F double-substituted variant, which was unable to perform protein-primed synthesis, completely lacked the protective activity (Figure 7C). The effects of individual substitutions are also in good agreement with the *in vitro* results: while the Y298F-substituted AbiA was strongly compromised *in vivo*, the Y303F variant retains a near-wildtype antiphage activity. Finally, HEPN-targeting substitutions R581A, H588A as well as the double R581A/H588A substitution had no effect on the antiphage activity, further supporting the structural role of the domain (Figure 7C).

### Antiphage activity of AbiK and AbiA likely does not rely on induced cell death

A standard test for a phage defense system acting by inducing the death (or stasis) of the virus-infected cell—abortive infection *sensu stricto*—are liquid culture infection assays with increasing multiplicity of infection (MOI), that is the ratio between phage particles and bacterial cells. Abortive defense systems are efficient at low MOIs (<1): while the minority of infected cells are killed by the triggered system, the majority of uninfected cells continue growth, thus ensuring the protection of the population level. However, at high MOIs (>1) efficient population-level abortive defense is impossible: as the majority of the cells are infected, they either succumb to the phage or are compromised by the action of the defense system.

We have performed liquid culture infection assays with increasing MOIs (0.1, 1 and 10) using *E. coli* BW25113 expressing AbiA or AbiK (*E. coli* transformed with the empty vector was used as the control). We tested the phages that the respective system provided protection against: six *Queuovirinae* representatives (Bas20–Bas25) in the case of AbiA as well as one *Queuovirinae* (Bas20) and two *Demerecviridae* (Bas32 and Bas34) in the case of AbiK (Figure 7D, E, Supplementary Figure S9). While the effects varied between individual phages and defence systems, none of the experiments were indicative of abortive infection *sensu stricto*. Even at the MOI of 10, AbiA-expressing *E. coli* were virtually insensitive to Bas20 and Bas25. In other cases, we observed collapse or growth inhibition of the AbiA/AbiK-expressing cultures at high MOI (10 and 1), but the effect was smaller or delayed in comparison to the empty vector control cultures. Furthermore, there is no relative growth defect in AbiA/AbiK-expressing cultures prior to culture collapse, which would be indicative of AbiA/AbiK-induced cell death or stasis.

## Discussion

In this study, we describe the first structure of a poorly characterized bacterial Abi polymerase AbiA that is involved in bacterial antiviral defense. The structure reveals several elements that are similar to other Abi polymerases, such as the helical domain, and other antiviral proteins, such as the HEPN domain. However, AbiA also exhibits features that are unique among Abi polymerases. In particular, unlike trimeric/hexameric AbiK and Abi-P2 enzymes (25), AbiA forms dimers.

The analysis of intersubunit contacts with PDBePISA (44) indicated that the average interface area between two protein chains within a dimer is 2172 Å^2^. According to PDBePISA calculations, the AbiA dimer should be stable in solution, in contrast to a tetramer. In comparison, for AbiK, PDBePISA predicts that both trimers and hexamers are stable oligomeric states, which is in agreement with experimental data (25). We compared the interface between two dimers in AbiA to the interface between the two trimers of the AbiK hexamer. AbiK trimer/trimer interactions are mediated by its RT-like domain (helices α9 and α11, α12/α13 intervening loop, helix α14, and strand β1) and by its helical domain (loops between α8’/α9’ and α12’/α13’) (25). The helices α9 and α14 in AbiK have clear counterparts in AbiA, but α11 does not. Moreover, helical domains of AbiK and AbiA are overall similar in shape, but the particular helices are not superimposable. The helical domain of AbiA is also significantly smaller, comprising 12 helices (the AbiK helical domain comprises 16 helices) and ∼170 residues (the AbiK helical domain consists of ∼300 residues). Overall, interactions that stabilize the AbiK hexamer involve elements that are absent in the AbiA structure, which might be why the AbiA tetramer is unstable in solution.

To investigate the function of AbiA dimerization we worked on preparing a monomeric form of the enzyme. To this end we introduced tryptophan substitutions that would disrupt the extensive dimer interface, comprising regions from both RT and HEPN domains. We have prepared the following variants: L515W, Y80W/T269W, Y80W/T269W/L515W and L162W/R601A. However, all of them formed dimers as assessed by GF/MALS (data not shown). Therefore, we were unable to generate a variant of AbiA which would be monomeric and we could not assess the importance of the dimerization of the full-length protein on activity. We note that ΔHEPN mutant which is monomeric is inactive. However, such a large deletion might introduce defects other than dimer disruption. The analysis of AbiA structure indicates that it is the same subunit of the dimer that performs DNA polymerization and protein priming. Any conformation/trajectory of DNA in which it is attached to Tyr298 or Tyr303 of one subunit and runs toward the active site of the other subunit to continue DNA synthesis seems unlikely, as the loop that harbors priming tyrosines could not reach to the active site of the other subunit of AbiA dimer.

The polymerase activity assays confirmed the ability of AbiA to *in vitro* synthesize long ∼150 nt ssDNA products. Wild-type, full-length AbiA was co-purified with 10–40 nt DNA fragments and 5–9 nt of the DNA chain could be traced in respective subunits of the crystal structure. Incubation with the dNTP mix resulted in the rapid emergence of longer products. AbiA polymerization products in a bacterial cell may be longer but are mostly degraded by viscolase that is used in the purification procedure. It is important to note that the protein already prior to starting the enzymatic assays is associated with co-purified fragments of the DNA of different lengths. Furthermore, the reaction products are heterogeneous in length (they migrate as a smear, see Figures 5 and 6). Finally, both Tyr298 and Tyr303 can be used for priming with different efficiencies of DNA extension. Therefore, precise estimation of AbiA processivity is challenging, and we can only provide a rough estimate of initial polymerization rate that is > 40 nucleotides/minute (Supplementary Figure S10). Importantly, all experiments were performed with AbiA that was not ‘triggered’ by the phage infection. Therefore, both the substrate preferences and the catalytic efficiently of the activated AbiA remain unknown.

The key aspect of the Abi polymerase mechanism is protein priming, in which the protein covalently attaches the first nucleotide to one of its own residues (hydroxyl-containing tyrosine, serine, or threonine). In our structure, we did not observe an electron density for the first nucleotides of the nascent DNA, so the priming residue could not be visualized or identified directly. Priming residue in AbiK has been previously identified as a tyrosine positioned in a loop that connects helices in N-terminal regions of the fingers subdomain (25). In the crystal structure of AbiA that is described herein, a loop that connects the RT-like domain and the helical domain and comprises residues 290–310 is not visible and thus can be assumed to be flexible. The structural analysis also indicated that conformational rearrangements of this region could allow residues within the 290–310 loop to move into the polymerase active site. There are two tyrosine residues within this loop - Tyr298 and Tyr303, and the former is evolutionarily conserved (Supplementary Figure S11). To test the importance of the tyrosine residues in polymerase activity, we prepared a Y298F, Y303F and Y298F/Y303F variants of AbiA and tested their activity. Analysis of the reaction products showed that single substitution variants maintain polymerase activity while the double-substituted variant has lost it. This suggests that both tyrosines can be used for protein priming. To our knowledge, this is a unique property of *L. lactis* AbiA. We note that only Tyr298 is conserved among AbiA proteins from multiple species (Supplementary Figure S11), so this is likely the residue universally used by AbiA proteins. Its substitution with phenylalanine produced a stronger defect both *in vitro* and *in vivo* than the corresponding substitution of Tyr303.

It would be interesting to establish whether AbiA and other Abi RTs are able to perform DNA synthesis *de novo* and without the use of protein-priming. Such primase activity has recently been reported for several members of the RT family, including CRISPR associated RTs, group II intron RTs, and HIV-1 RT (56). Primer-independent DNA synthesis has also been observed for piPolB enzymes, a subgroups of family B DNA polymerases found in self-replicating mobile genetic elements called pipolins (57). Moreover, there are known RNA polymerases that are able to synthesize the product chain *de novo*, such as QDE-1 from *N. crassa* (58). RDR2 and RDR6 from *A. thaliana* have also been shown to initiate nucleic acid synthesis in both primer-dependent and primer-independent modes (59). Both modes of initiation of RNA synthesis are also observed for some of the viral RdRPs, including the enzymes from flaviviruses (60). These are RNA polymerases, and their architecture differs significantly from RTs, but the dual mode of synthesis initiation itself could be similar for AbiA.

Our finding that expression of AbiA protects *E. coli* from phages in the *Queuovirinae* subfamily provided a system to investigate *in vivo* role of key residues in the RT-like and HEPN domains. As expected, the DNA polymerase activity of AbiA is required for antiphage defense. Consistent with the notion that Tyr298 is the key priming residue, the Y298F AbiA variant is strongly compromised in phage protection whereas the Y303F variant retains most of its antiphage activity. Moreover, substitutions in the R-X_(4–6)_-H motif (R581A, H588A or R581A/H588A) of the HEPN domain did not influence the antiphage activity, indicating that the presumed nuclease activity of AbiA is not required at least for defense against the phages tested in this study. Both AbiA and AbiK have been suggested to be abortive infection systems (18,20) and as such they are expected to induce cell death upon phage infection. However, in our liquid phage infection experiments, neither system appeared to induce abortive infection *sensu stricto*. Although it cannot be excluded that the apparent lack of abortive infection is due to low infectivity of the tested phages, the observations imply that AbiA and AbiK may not be *bona fide* abortive infection systems. In fact, recent experiments in *Lactococcus lactis* where AbiK^+^ and AbiK^−^ strains were challenged with high MOIs of lactococcal phage p2 suggested that the antiphage defense mediated by AbiK may not necessarily involve the triggering of cell death (26).

In conclusion, we provide structural, biochemical and microbiological insights into previously uncharacterized molecular workings of the AbiA subfamily of Abi polymerases. Given the current research interest in the antiphage defenses mediated by UG/Abi RTs (17), the biochemical data and structure that are presented herein are a valuable addition to the field of DNA polymerase structure and function research.

## Supplementary Material

gkae230_Supplemental_Files

## Data Availability

Atomic coordinates and structure factors for the reported AbiA crystal structure were deposited in the Protein Data Bank (PDB; accession no 8OZ7).
