# Supplementary material for: Structure-functional characterization of Lactococcus AbiA phage defense system
Source: Nucleic Acids Res. 2024 Apr 8;52(8):4723–38. doi: 10.1093/nar/gkae230 (PMC11077055; doi:10.1093/nar/gkae230)
Supplement: gkae230_Supplemental_Files [file gkae230_supplemental_files.zip › AbiA Supplementary.pdf]

SUPPLEMENTARY INFORMATION

Structure-functional characterization of *Lactococcus* AbiA phage defense system

AUTHORS

Marta Gapińska[1,&], Weronika Zajko[1,&], Krzysztof Skowronek[2], Małgorzata Figiel[1], Paweł S. Krawczyk[3], Artyom A. Egorov[4], Andrzej Dziembowski[3], Marcus J. O. Johansson[4,*], Marcin Nowotny[1,*]

[1] Laboratory of Protein Structure, International Institute of Molecular and Cell Biology, Warsaw, Poland
[2] Biophysics Core Facility, International Institute of Molecular and Cell Biology, Warsaw, Poland (RRID:SCR_021630)
[3] Laboratory of RNA Biology, International Institute of Molecular and Cell Biology, Warsaw, Poland
[4] Department of Experimental Medical Science, Lund University, 221 00 Lund, Sweden

& contributed equally

* To whom correspondence should be addressed:

M.N.: Tel: +48 22 5970717; Email: mnowotny@iimcb.gov.pl

M.J.O.J.: Tel: +46 73 021 48 68; Email: marcus.johansson@med.lu.se

Supplementary Table 1. Phage clustering based on proteome similarity.

The table is provided as a separate Excel file.

Annotation of BASEL and common laboratory phages using current taxonomy information (Family, Subfamily, Genus) extracted from NCBI Taxonomy database (41, January 2024 update) and ICTV (42, 2022 update) (“Annotation”, sheet 1); The phage clustering results (“Proteome clustering”, sheet 2).

Supplementary Table 2. Crystallographic data collection and refinement statistics.

	**AbiA**
**Data collection**	
Space group	*P*1
Cell dimensions	
a, b, c (Å)	84.1, 86.1, 117.0
α, β, γ (°)	99.3, 90.2, 90.3
Resolution (Å)	38.49-2.75<br>(2.92-2.75)
$I/\sigma I$	9.03 (1.11)
$CC_{1/2}$	99.6 (56.1)
Completeness (%)	95.2 (93.3)
Multiplicity	3.65 (3.57)
**Refinement statistics**	
Resolution (Å)	38.49 - 2.75
No. of reflections	80156
R_{work} (%)	22.0
R_{free} (%)	26.4
No. of atoms	19626
macromolecules	19512
ligands/ions	1
water	113
B factors (Å)	
macromolecules	72.53
ligands	84.09
Root mean square deviations	
Bond lengths (Å)	0.002
Bond angles (°)	0.44

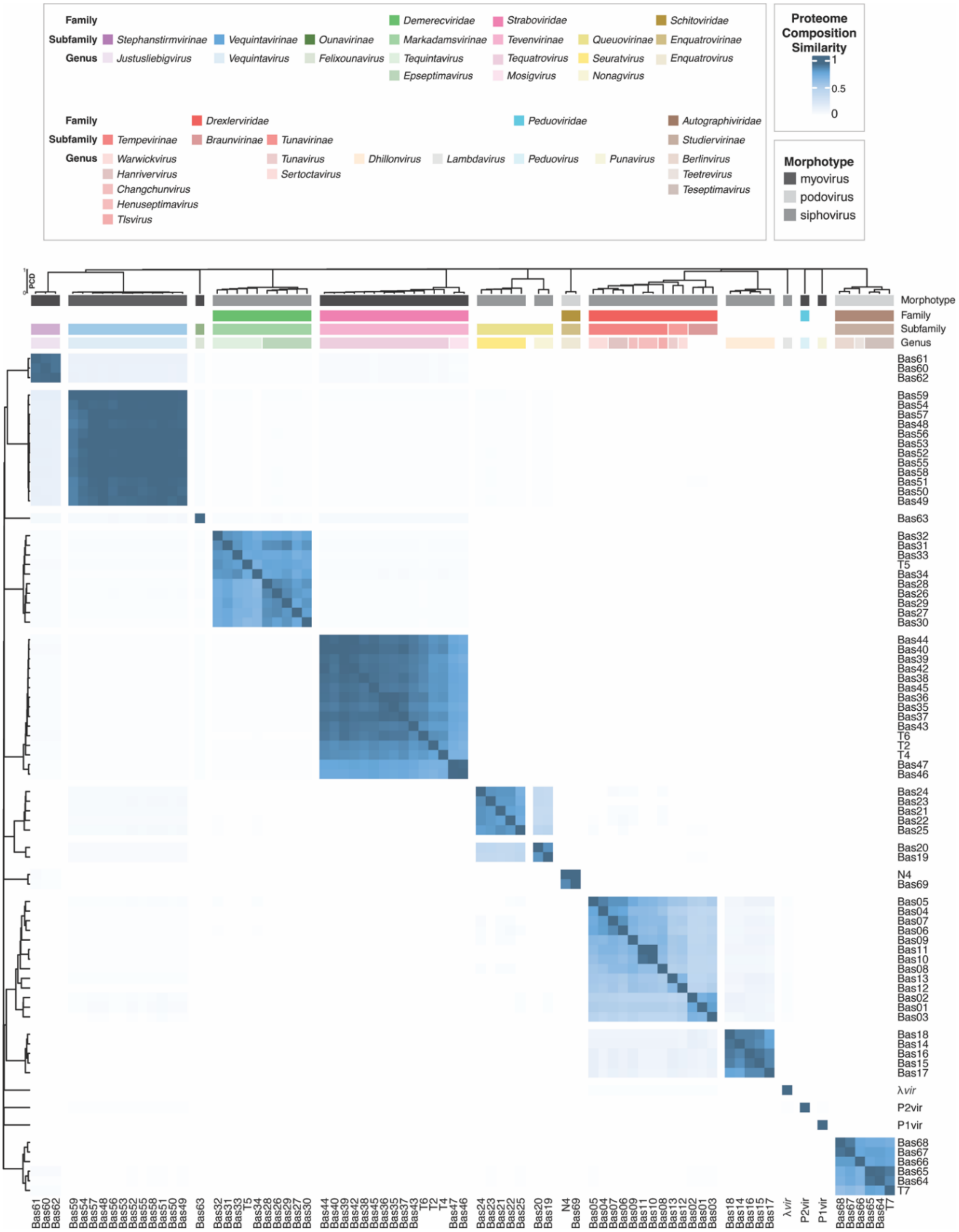

Supplementary Figure S1. Hierarchical clustering of BASEL and common laboratory coliphages using pairwise proteome similarity scores. To cluster the phages on the basis of proteome similarity, the number of shared homologous proteins between all phage pairs were computed and normalised to the phage proteome length, and the matrix of these pairwise similarity scores were used to compute the complement symmetric proteome composition distance (PCD) matrix. Hierarchical clustering using the average-linkage method applied to the PCD matrix was used to generate the dendrogram that determines the phage groupings. Phage annotations followed the latest ICTV update (42).

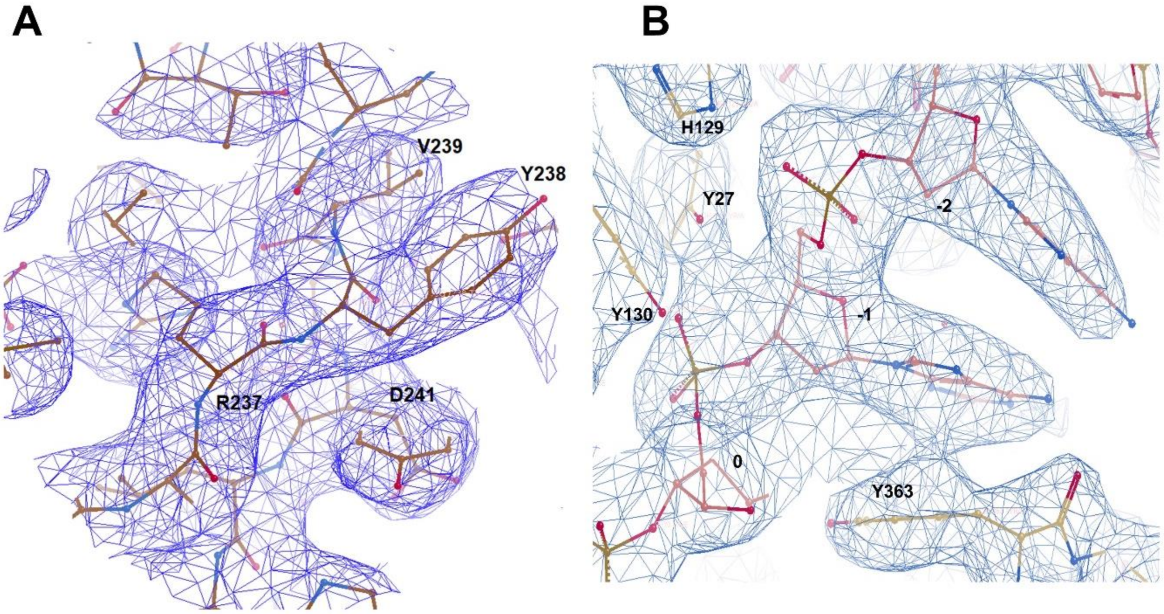

Supplementary Figure S2. **AbiA electron density maps.** Simulated annealing composite omit electron density maps contoured at 1.0 σ overlaid on fragments of the structure around the active site (**A**) and around the DNA product (**B**).

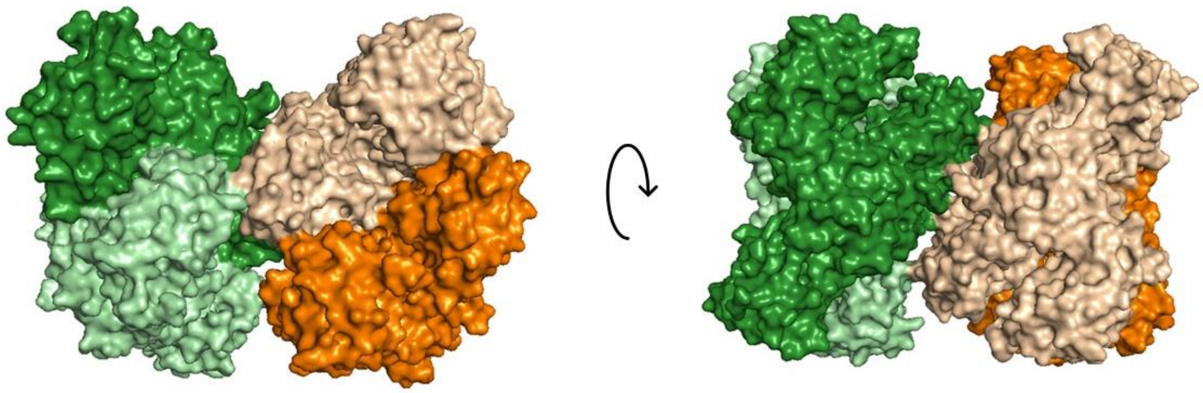

Supplementary Figure S3. **Tetramer of AbiA (two views).** Subunits within one dimer are shown in shades of green, and subunits within the other are presented in shades of orange.

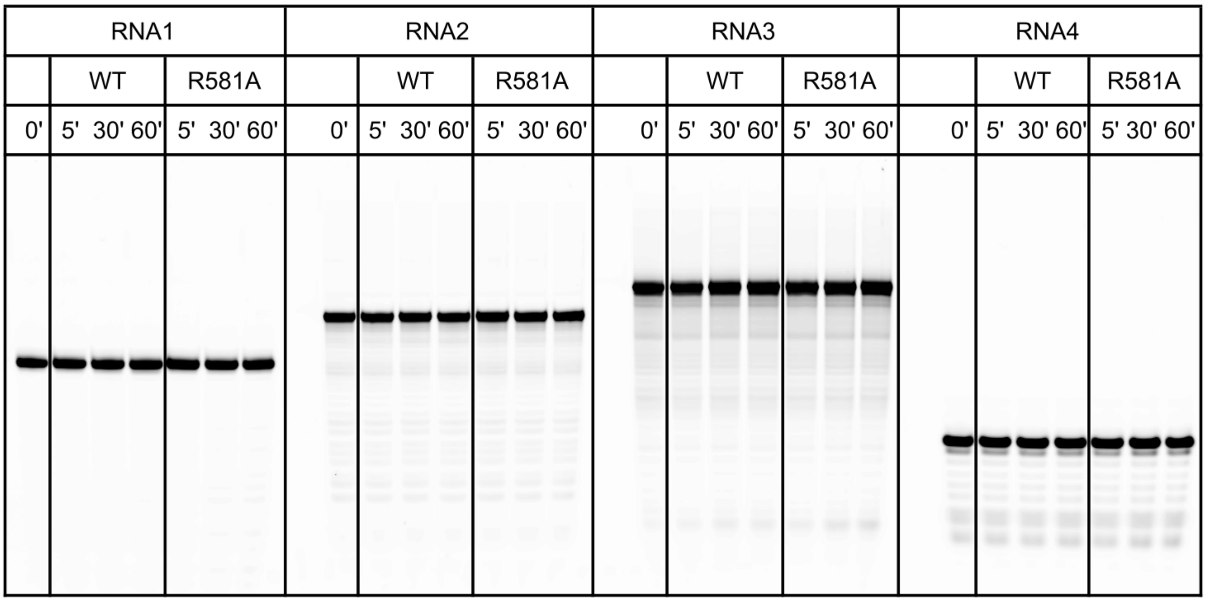

Supplementary Figure S4. Nuclease activity assays with wild-type AbiA and its R581A point substitution variant. 1 μM of wild-type or HEPN active site point substitution R581A variant AbiA protein was incubated at 37°C with 1 μM fluorescent-tagged RNA substrates for 5, 30, and 60 minutes in a reaction buffer consisting of 50 mM Tris (pH 7.0), 250 mM NaCl, 10 mM MgCl2, and 5 mM DTT (see Materials and Methods for sequences). The reaction was then stopped by the addition of 40 mM EDTA and proteinase K. Reaction products were then resolved on 20% denaturing TBE-urea polyacrylamide gels and visualized by fluorescence readout with the Amersham Typhoon (Cytiva) biomolecular imager.

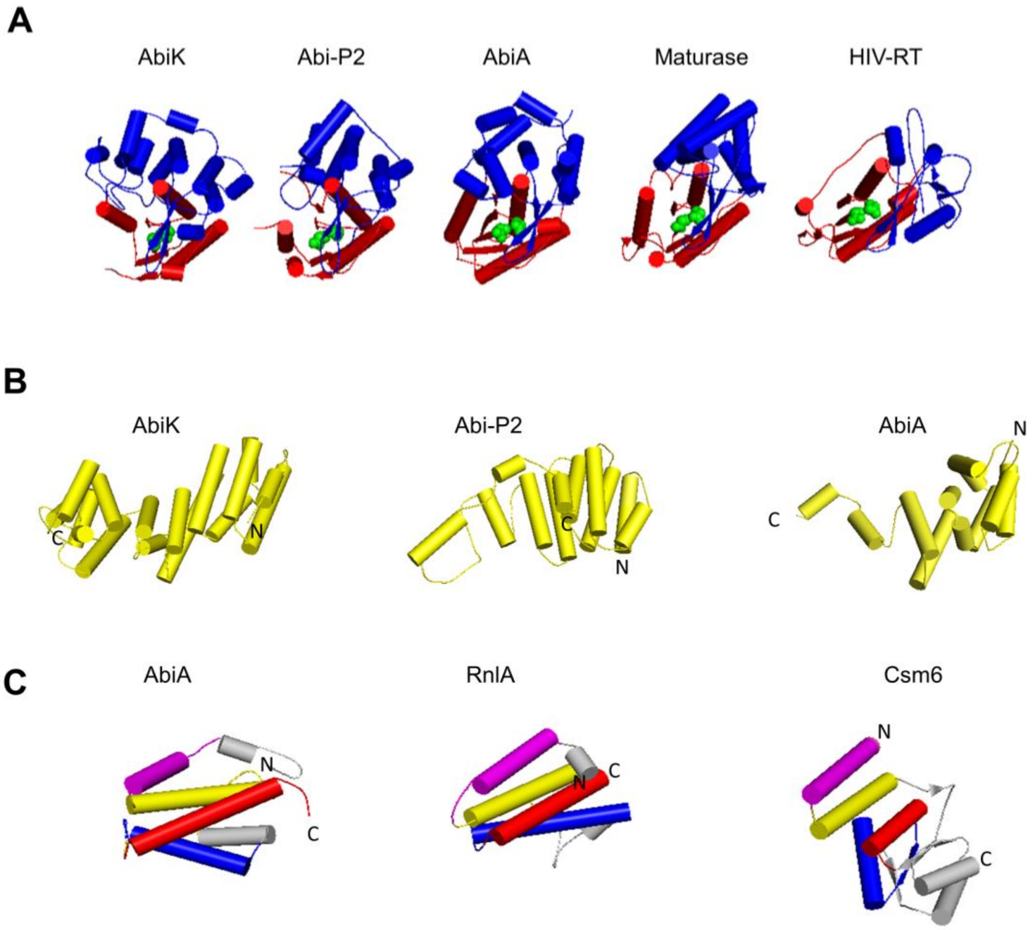

Supplementary Figure S5. Structural comparison of AbiA with other RT proteins. (**A**) Comparison of RT-like domains. From *left* to *right*: RT-like domains of AbiK (PDB ID: 7R07), Abi-P2 (PDB ID: 7R08), AbiA, group II intron maturase (PDB ID: 6AR1) and human immunodeficiency virus 1 (PDB ID: 4PQU). Blue indicates fingers subdomain, red indicates the palm subdomain. Two evolutionarily conserved aspartic acid residues from the RYVDD motif in the active site are shown as green spheres. (**B**) Comparison of helical domains from AbiK (PDB ID: 7R07), Abi-P2 (PDB ID: 7R08) and AbiA. (**C**) Comparison of HEPN domains from: AbiA, RnlA toxin from the toxin-antitoxin RnlAB system (PDB ID: 6Y2Q) and Csm6 protein that is involved in CRISPR (PDB ID: 5FSH) and. Corresponding helices are shown in the same color.

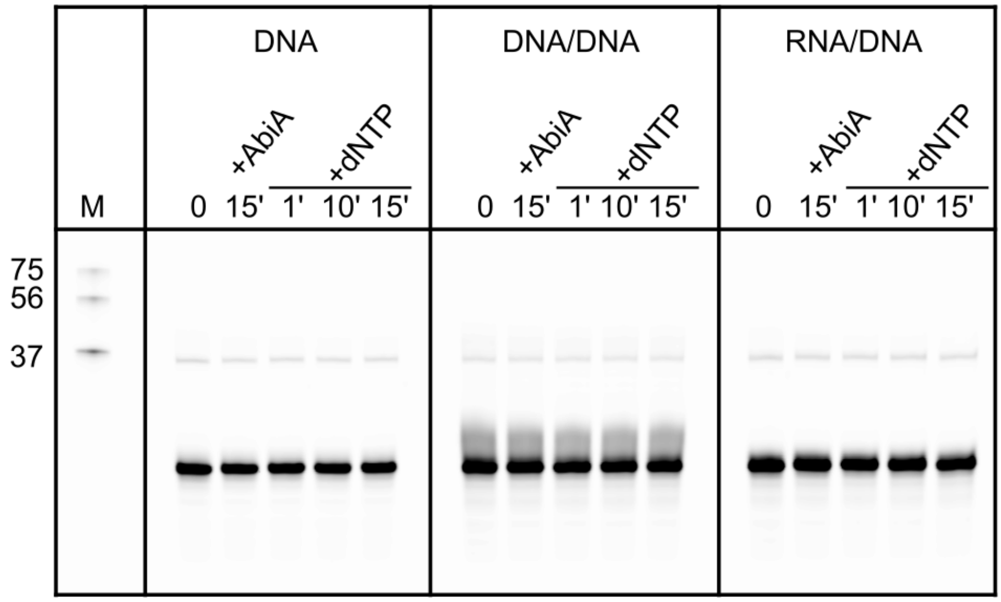

Supplementary Figure S6. External primer extension assay. 0.5 µM Cy5-5′-labeled primer (see Materials and Methods for sequences), was incubated at 37°C for 15 minutes with 0.1 µM AbiA Y298F/Y303F variant. Then dNTP mix was added at a concentration of 250 µM, and the reaction was further incubated for 1, 10, and 15 minutes. The samples were treated analogously to the polymerization assays (see Figure 5). Similarly, reaction was performed in the presence of a hybrid which contains a labeled DNA primer with a DNA or RNA template in molar ratio 1:1.5.

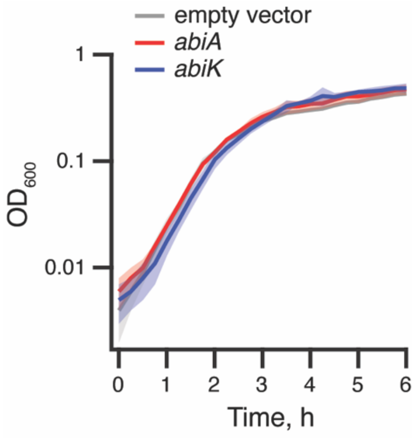

Supplementary Figure S7. The growth of the *E. coli* K-12 strain BW25113 is not affected by constitutive AbiA or AbiK expression. Cultures from three different transformants of each plasmid (empty vector (pJD1423) or the same plasmid carrying the *abiA* or *abiK* gene under the P_{tet} promoter) were back-diluted in LB+Amp (100 µg/ml) medium and the growth was monitored at 37°C in a Synergy H1 (BioTek) plate reader by measuring OD_{600} every 15 min.

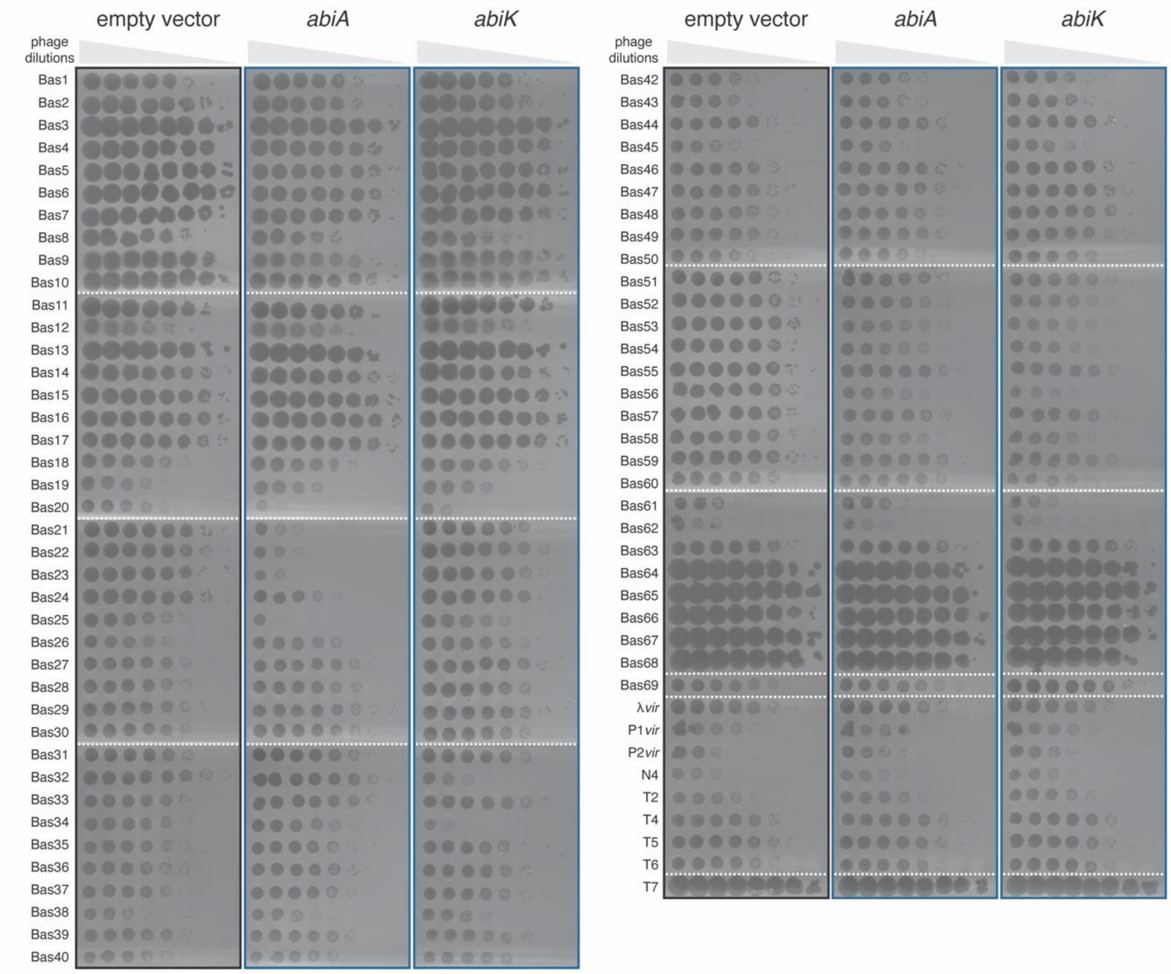

Supplementary Figure S8. Expression AbiA or AbiK in the *E. coli* K-12 strain BW25113 grants protection to a subset of BASEL coliphages. Plaque assays on lawns of *E. coli* BW25113 harboring the empty pBR322 derivative pJD1423 or the same plasmid expressing AbiA (VHp1642) or AbiK (VHp1639) from the P_{tet} promoter. The indicated phages were 10-fold serially diluted and spotted on the top agar plates.

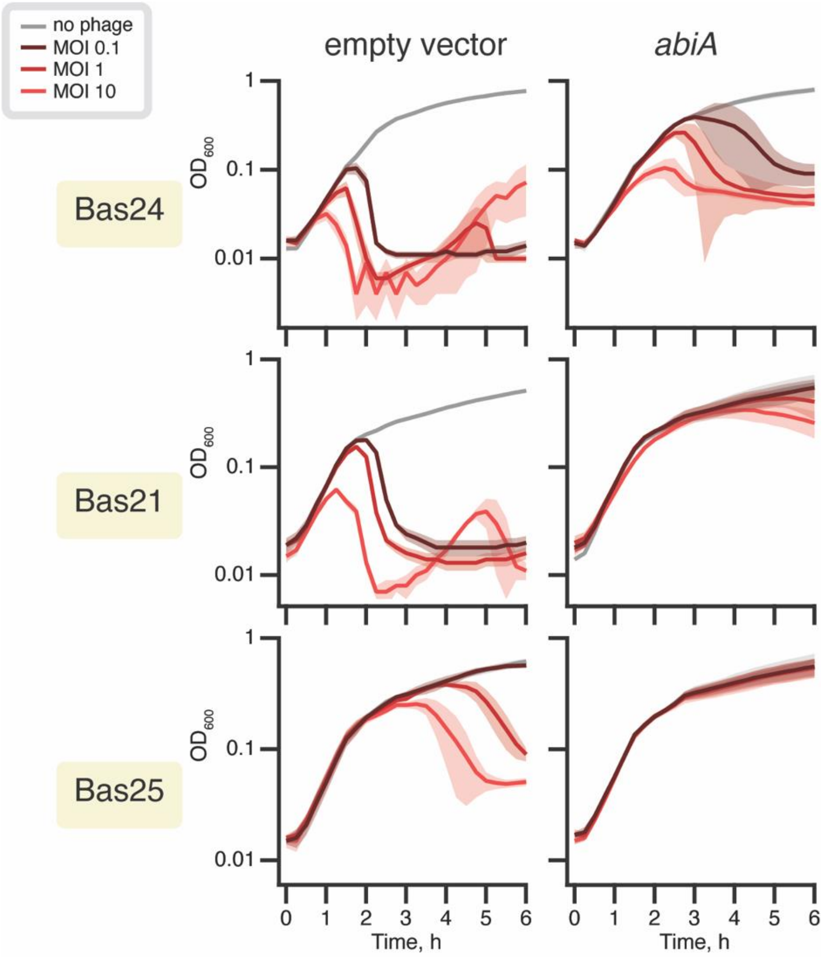

Supplementary Figure S9. Antiphage defense by AbiA in liquid *E. coli* cultures. Growth of BW25113 carrying the empty vector or a plasmid-encoded *abiA* gene in the presence of indicated phages at MOIs of 0, 0.1, 1 and 10. The curves represent the average of three replicates, using different transformants for each replicate, and the shaded areas indicate the standard deviation.

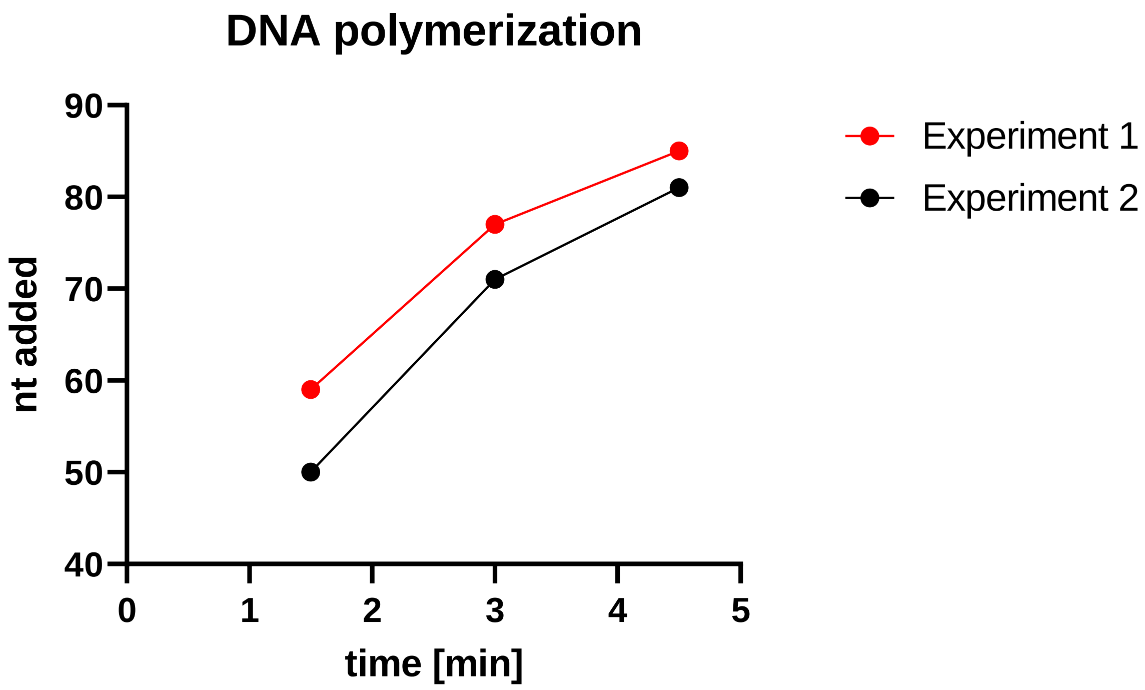

Supplementary Figure S10. AbiA DNA polymerization rate. Average product size (in nucleotides) is plotted vs reaction time. Results of two repetitions of the experiment shown in Figure 4 are presented. Average sizes of the labeled primers (at time 0) were 17 and 15 nt for experiment 1 and experiment 2 respectively.

```
Globicatella_sanguinis       1   -------MSKSTWFEVVNKYFDLS-KGKLSKYLQWYPF--TKKSVRKELLSDDFYNHIIKSGYFLFDKQVMEVTSNYIQ   69
Staphylococcus_aureus        1   ----MININFNEWEKVCKSFFKLN-KSILESYLQWYPFSKLTENSKCTILSEKFFFNFIKNGAIFKEYNTFNFPSHYSQ   74
Oceanobacillus_oncorhynch    1   ----MINMEYDTWNNVCEGIFKQS-KQSLNKYLQLYPFSILTDDEKKLIKSEEFFYKFINNGALFKNKLVFDFPRHYIQ   74
Lactobacillus_apis           1   MNNLLLGFNYNNWKRTCNLFLNPKIKRSLSMYLQDYPLAKLSSENIETIKSEDFFNEYIETGKVFFNSTVIWRTSNFIT   79
Lactococcus_lactis           1   ----MITLQHQDWERAVNMIKNIP-PSAKNKYFQTFPFFLLSETSWEELLSENFFYSYIKSGEFLTYQENLSFYDRTIQ   74
Consensus_aa:                    ....hlshp.psWbchhp.hbp......bsbYhQ.@Ph.bhppps.cplbS-pF@.phIcsG.hh..ppsh...sphIp

Globicatella_sanguinis      70   KSDGTYRQSQLLSPLLSMILESICKEI-FILIDGKLNN--SHALYAGDLG--DNSVTYSKQYNLFYKEVNKLAGIFPFY   143
Staphylococcus_aureus       75   KTSASFRDMTLVSPFVYLYIEVVGYHISKKYTRK-SKY--VRCYYSGDLS--ENEFSYKNSYDKFFADINALSSTYDNF   148
Oceanobacillus_oncorhynch   75   KNNSSFRNSKLVSPIIYIYLECIGYHVCKAYIKDSTTT--RCYYAGNIHE---LDFHYQKSYERYYADVNECSQRYRYY   148
Lactobacillus_apis          80   KNSGGFRDAQLLSPFLFLLLQAIGVEIQSRYVPLRSKNPKLIAYYSGDFS--RNIVTYKSQYRGFCEVNTQLSKEFPYF   156
Lactococcus_lactis          75   KSHGAYRQTRIVSPIIYIFLIAIASQVERIYVEKRTND--MSVYFSGSFEKEKNTAHYKQSYNTYMTELNACQEEFDYY   151
Consensus_aa:                    Kspts@Rpspll SPhl.hhLbtIt.pl...hl..p.ps....hh@tGsh...cN.hhYpppYp.@h...s.h...@s@@

Globicatella_sanguinis     144   MKFDINNFFRNIDINLLFSMIEEKTNKIPQFHIYIYKQFFEFI-------GQGKFPVVENSTGLSYLATKIYLNDIDEE   215
Staphylococcus_aureus      149   YKFDISNFFDAVDINLLFKLINEGEEILDTRSSLIYKRLLQQI-------GGNKFPTLENSSTLSYLATYIYLDKVDYE   220
Oceanobacillus_oncorhynch  149   YKFDVTNFFDSLDINILFENINKKSTIINSRTALIYKRLLQSI-------GSGKYPTIEKSCSLSFLATYVYLDLFDSK   220
Lactobacillus_apis         157   IKTDISDYFSNINLDRLLAMINSR--IDKENSSKFTPLQLKMIKEMLYYCGNGKFPLVENSTCSSFLATIIYLDEIDDK   233
Lactococcus_lactis         152   FQTDFSTFFHLVDTDNLFNKIDRL----DPKSALVYSSLIKMI-------GQGRMPIVDGNSGLSFLNTVVYLDDFDKE   219
Consensus_aa:                    hphDhss@Fp.lshs.Lh.bIpp...b....p.bhh..bhpbI.......GpG+hPhl-ssot.S@LsT.lYLschDpc

Globicatella_sanguinis     216   IN-NFFIRENITMFTFIRYVDDLYIFID------PEIDGVNPEKLFNKFKHFYT---TNLRDLNLNINSQKS-TLNESK   283
Staphylococcus_aureus      221   LEKVLQKNSKIESFQIIRYVDDLYIFFN---TMESELNLVS-SEIKNVVIDAYR-------KVKLNLNENKT-KLGKSS   287
Oceanobacillus_oncorhynch  221   LEGFLGDLHDIVDFQIIRYVDDLFIFFN---TSEDLLNEVV-SIIKNFVIHSYR-------EVNLNLNEQKS-NYGFSN   287
Lactobacillus_apis         234   MGSFIDDMTDIEDYRLVRYVDDLYIWIKPKDYQNQDKEDYN-SEYNNIRFKYSSLLH----SYGLTLNTNKT-LFCKSE   306
Lactococcus_lactis         220   IIDSLKTIVEIESFKLVRYVDDLHIFIK---CANKDLDFLN-YKVYNLLCEKAT-------KHHLEINSSKTKSFTPTS   287
Consensus_aa:                    h...h....pIp.@phlRYVDDL@I@hp...h.p.-bp.hs..ch.Nb..c..p.......ph.LplNppKo..hs.op

Globicatella_sanguinis     284   DINQSLKKSFYMGEIYEEDINLHDIPFQKYEL--FLKKLVENLES----NGTISIDDYDKIKNEVFNWPDIELS-----   351
Staphylococcus_aureus      288   EVNETLSVALYNHYVYKEEIDIAHF-YDKNKILLFLDDL-YNLAY----SHNH--ENFKYILDKHFTKEGITYS-----   353
Oceanobacillus_oncorhynch  288   NISEELGAALYDHYVNEQDIDIPSM-FDENNIKVFVDKL-YEIARVRLHNHDR--YREIKDEVFT--IPDVQYS-----   355
Lactobacillus_apis         307   GIFEQLKLNIYDEIIPEK----ENP-ISLNKE-EAVDKI-KNFM------SDL--INFNKKHNLTKEDLENIIDNNFRP   370
Lactococcus_lactis         288   ELSTKMNTDLYNFFVYNEDVDFEQY-FSKNTLIEFLDKL-NNMSV----NADF--SEYEKEVLYTLENPEIVSD-----   353
Consensus_aa:                    .l.pphp.shY..bl.pc-lsh.p..hpb.pb..hlcKl..Nh......s.sh...p@pKb..bhbp..-...s.....

Globicatella_sanguinis     352   -------VEEAYNTIVYNFSDEFINNTTIIDSL-NKLLEDEV--ILFDVKRFVVAILHT-KEGT---LIKKLLNTMF--   414
Staphylococcus_aureus      354   -------SDEVLRYLAFYED-ELFQDEVIIYKV-KRLILTDYNFVNYKINIFLRIILNT-NNGD---LIRYLLNELF--   417
Oceanobacillus_oncorhynch  356   -------SDEVFRYLIYYRK-GIFKDTNLIDKL-KRLIKRDYKIIKYDTKNLIYMILNT-RDEE---LIKHFLNEIF--   419
Lactobacillus_apis         371   KDAIDFTGEEVLRNIIFDKP-EYLNDGNIISSINKLIIERGISFIYLSPKLFTNMVLNTSRDQNRNKAVKTLLNEIFLQ   448
Lactococcus_lactis         354   -------GSYILNAIVYNKS-TWSQDYDIKNKI-SLLVNSNYRKLRYSAKALITLVLNT-RDGD---IIKGLLNNLFTT   419
Consensus_aa:                    .......spbhhp.ll@.bs.ph.ps.sIbspl.p.llpp.hpbl.hssK.hh.hlLpT.+-.s...hlK.LLNphF..

Globicatella_sanguinis     415   -RKKYWNDSDNYIAITYLLQRSFAHSDLKK-TLQLNLEDRNPGLFNYIEAYCKG--IKSWKR--PGNELFIKK--CKSY   485
Staphylococcus_aureus      418   -KKDNFNSFDVAISLNYLLLRNFQHTDLMY-KIRAV----DSDIIDYINRYCKQDFLKELDKEYNYIIKLNLN--REFS   488
Oceanobacillus_oncorhynch  420   -RNRGLDSFDIAMIINYLILRNFRHQDLMN-KLAEY----EPNIYKYINLYCKGSFVKSLLD--EDYNSYINLIFEDDY   490
Lactobacillus_apis         449   LRSGALNAYDINISLFYLLRTDFKHNDLKNKVIKEY----DPKLFEYIAMYCVKDFFCSFCEE-NRK--------KSIY   514
Lactococcus_lactis         420   FKNGTNDIIDEIILIEYLVQRKFNHKDLMT-ILKAD----DHGIKEYIKAYQTSDFIKSLEK--NKVIFYTNQ--KEVY   489
Consensus_aa:                    .+p...s..D..I.l.YLl.ppF.HpDLbp..lp......s..lbpYI.hY...sFh.Sh.c..s....h......p.Y

Globicatella_sanguinis     486   -NISTTTMHLYFLYLKAK---ADDDALLGHAYFKTLFDRITAELSYSFGIEVTKR--KSKKIVYNNFYEEKILSSIY--   556
Staphylococcus_aureus      489   -DSSL--K-VWYLYFLYKYYDKNGDTLEAFAYYKTYFDRMVSLLMYYKKISIGKKGSPNYKRHYK---VGNAQTDFEKL   560
Oceanobacillus_oncorhynch  491   -DFNNDLK-VWYIYFMYQHYKNSNDALEAFSYYKSYFDRVTALLMHYKGIEQTGRGKPNYKMFYK---QSQIINGYKRI   564
Lactobacillus_apis         515   LQVISSDWKTYYLYANYLFEQERNNKIEAFAYYKTYFDRFTA---LISAYTTKTK--LKVKDFYS---IEDLNKVYKDL   585
Lactococcus_lactis         490   -PLISKDKILNFIYFRAKYFESLDLVLESFAYYKNYFDRFVAHAMFCTGIDSGRK--PNYKLYYT---EGKLIDGLKQL   562
Consensus_aa:                    ..h.sp.b.h.@lYh.hb@bp..s..lbt@AY@KshFDRhhA.h.h..thp..p+...p.K.hYp...b.pl.pshbpl

Globicatella_sanguinis     557   ----GDYDSGGQIIKRAHKIRNSNPIIHSSSELAKGSR-TKE-------DIINSIEDLKKLIKEFIRDFTEP   616
Staphylococcus_aureus      561   NARYYKNKNVSTLLSELYKLRNFNPINHSSAEIIEDKMLKKS-------QITNLIKQAETLLFD---SFSS-   621
Oceanobacillus_oncorhynch  565   GID-QGAINIEDLITKAHDLRNHNPINHSSAEVMDHETLNLQ-------EIENIIGELELLLEQ---GFSV-   624
Lactobacillus_apis         586   E-----GINSYKIIKKANELRNENPLVHSSAKLIGRPSLE---------GIDVCIKQLQDLIKLRIESYD--   641
Lactococcus_lactis         563   NF--LSSDEITKIINEAHKIRNSNPVSHSSAGLLQNEDFSRYRVKSSLNDLKIIIEQLSTLLQN---KNRL-   628
Consensus_aa:                    ........p..plIpcApclRNpNPl.HSSt.lh...php..........l...IcpLppLlp....p.p..
```

Supplementary Figure S11. Multiple sequence alignment of representative AbiA sequences. Alignment was performed using Promals3D (61) based on five AbiA sequences. Sequences that correspond to α-helices are shown in blue, and sequences that correspond to β-strands are shown in red. The sequence of the polymerase that was studied herein is indicated in yellow. The evolutionarily conserved R-$X_{(4\text{-}6)}$-H motif that is associated with nucleolytic activity is highlighted in cyan. The evolutionarily conserved tyrosine (numbered 298 in AbiA from *L. lactis*) is highlighted in green.
